# Downregulation of hsa-microRNA-204-5p and identification of its potential regulatory network in non-small cell lung cancer: RT-qPCR, bioinformatic- and meta-analyses

**DOI:** 10.1186/s12931-020-1274-9

**Published:** 2020-02-26

**Authors:** Chang-Yu Liang, Zu-Yun Li, Ting-Qing Gan, Ye-Ying Fang, Bin-Liang Gan, Wen-Jie Chen, Yi-Wu Dang, Ke Shi, Zhen-Bo Feng, Gang Chen

**Affiliations:** 1 Department of Pathology, First Affiliated Hospital of Guangxi Medical University, Nanning, 530021 Guangxi Zhuang Autonomous Region People’s Republic of China; 2 Department of Medical Oncology, Second Affiliated Hospital of Guangxi Medical University, Nanning, 530007 Guangxi Zhuang Autonomous Region People’s Republic of China; 3 Department of Radiotherapy, First Affiliated Hospital of Guangxi Medical University, Nanning, 530021 Guangxi Zhuang Autonomous Region People’s Republic of China

**Keywords:** miRNA-204-5p, NSCLC, Real time -qPCR, microRNA microarray, microRNA-sequencing, Molecular mechanisms

## Abstract

**Background:**

Pulmonary malignant neoplasms have a high worldwide morbidity and mortality, so the study of these malignancies using microRNAs (miRNAs) has attracted great interest and enthusiasm. The aim of this study was to determine the clinical effect of hsa-microRNA-204-5p (miR-204-5p) and its underlying molecular mechanisms in non-small cell lung cancer (NSCLC).

**Methods:**

Expression of miR-204-5p was investigated by real-time quantitative PCR (RT-qPCR). After data mining from public online repositories, several integrative assessment methods, including receiver operating characteristic (ROC) curves, hazard ratios (HR) with 95% confidence intervals (95% CI), and comprehensive meta-analyses, were conducted to explore the expression and clinical utility of miR-204-5p. The potential objects regulated and controlled by miR-204-5p in the course of NSCLC were identified by estimated target prediction and analysis. The regulatory network of miR-204-5p, with its target genes and transcription factors (TFs), was structured from database evidence and literature references.

**Results:**

The expression of miR-204-5p was downregulated in NSCLC, and the downtrend was related to gender, histological type, vascular invasion, tumor size, clinicopathologic grade and lymph node metastasis (P<0.05). MiR-204-5p was useful in prognosis, but was deemed unsuitable at present as an auxiliary diagnostic or prognostic risk factor for NSCLC due to the lack of statistical significance in meta-analyses and absence of large-scale investigations. Gene enrichment and annotation analyses identified miR-204-5p candidate targets that took part in various genetic activities and biological functions. The predicted TFs, like MAX, MYC, and RUNX1, interfered in regulatory networks involving miR-204-5p and its predicted hub genes, though a modulatory loop or axis of the miRNA-TF-gene that was out of range with shortage in database prediction, experimental proof and literature confirmation.

**Conclusions:**

The frequently observed decrease in miR-204-5p was helpful for NSCLC diagnosis. The estimated target genes and TFs contributed to the anti-oncogene effects of miR-204-5p.

## Background

The worldwide morbidity and mortality of pulmonary cancer has remained high for decades in both genders, reflecting an increase in contributory factors like tobacco use and air pollution [1–5]. The two primary categories of pulmonary neoplasms are small cell lung cancer (SCLC) and non-small cell lung cancer (NSCLC), with NSCLC accounting for approximately 80% of all pulmonary cancers. NSCLC includes adenocarcinoma, squamous cell lung carcinoma, undifferentiated large cell carcinoma, adenosquamous carcinoma and bronchioalveolar carcinoma; the first three are the best known types [6]. The survival of patients with NSCLC is still bleak due to delayed diagnosis, undisciplined treatment, incident chemoresistance, and frequent tumor recurrence [7–9]. Thus, thorough investigation of the molecular mechanisms underlying lung carcinogenesis remains an urgent task, for establishing new and effective guidelines for cancer screening and for identifying novel genetic targets for treatments.

One potential class of molecular targets are the microRNAs (miRNAs). These are small non-coding RNA molecules, with approximately 20 nucleotides in length, that negatively modulate expression of target genes by completely or incompletely binding to the 3′ untranslated region (UTR) of messenger RNAs (mRNAs) [10–13]. The miRNAs have been proposed as novel diagnostic biomarkers and prognostic indicators for tumorigenic processes, as they play indispensable roles in cancer cell differentiation, proliferation, and apoptosis, and in metastasis and recurrence of numerous malignant tumors [10, 14]. One miRNA, hsa-microRNA-204-5p (also known as miR-204-5p, or miR-204), has attracted attention in NSCLC research, because its low expression in NSCLC tumors is associated with advanced progression, poor prognosis and severe metastatic potential [15–17].

Previous studies on the mechanisms of miR-204-5p on NSCLC has mainly focused on the repression of specific mRNAs, so knowledge about its multilateral functions or its clinical prospects remains limited. Aberrant expression of miR-204-5p is now a well-established feature of pulmonary carcinogenesis; however, what is still unclear is the clinical contribution of miR-204-5p and particularly its potential role in the early detection of NSCLC. The mechanism by which miR-204-5p mediates its target mRNA-protein signaling networks to regulate tumor progression is also not yet established.

**Fig. 1:**
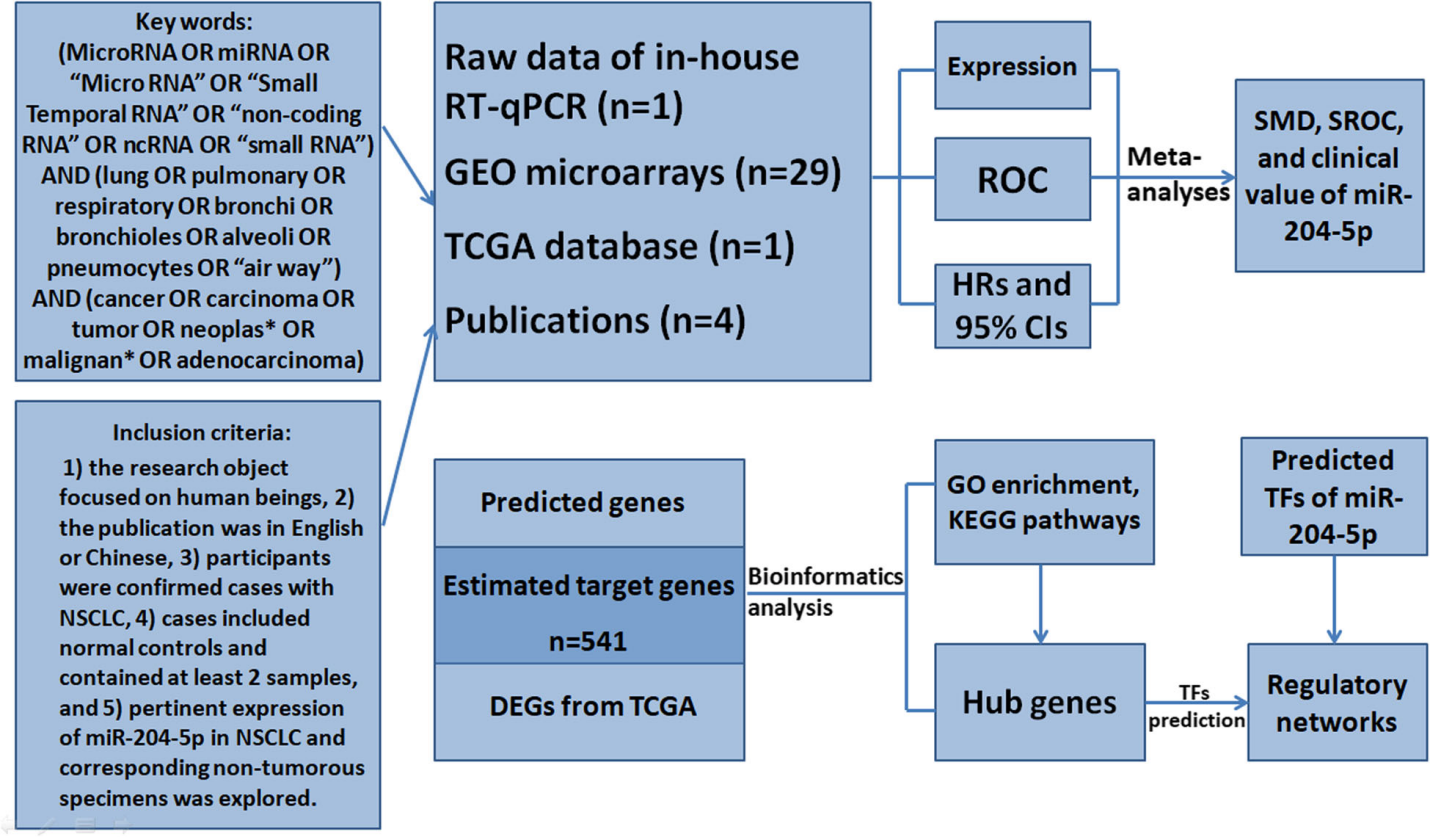
Study structure and major research methods in this paper. Abbreviation: GEO, Gene Expression Omnibus; TCGA, the cancer genome atlas; RT-qPCR, real-time quantitative polymerase chain reaction; ROC, receiver operating characteristics; HR, hazard ratio; 95% CI, 95% confidence interval; SMD, standard mean deviation; SROC, summarized receiver operating characteristics; DEGs, differentially expressed genes; GO, gene ontology; KEGG, Kyoto Encyclopedia of Genes and Genomes; TF, transcription factor

The current work describes distinctive features of miR-204-5p expression in NSCLC by integrative analysis of results from real-time quantitative polymerase chain reaction (RT-qPCR) and from sequence and genechip data from the cancer genome atlas (TCGA), Gene Expression Omnibus (GEO), and the current literature, in addition to relevant prediction materials from online tools. Our goals were to explore the possibility that miR-204-5p might be a promising indicator for NSCLC process and to identify our perspective on other underlying regulatory mechanisms at the molecular level (Fig. 1).

## Methods

### Patients and samples

Formalin-fixed, paraffin-embedded (FFPE) samples and corresponding non-cancerous lung tissues were obtained with prior informed consent from125 patients with NSCLC treated at Department of Pathology, the First Affiliated Hospital of Guangxi Medical University (Nanning, Guangxi, China) from January 2012 to February 2014. The research proposal was approved by the Committee on Ethics of the First Affiliated Hospital of Guangxi Medical University. All cases were pathologically distinguished and verified by two recognized experts (Zhen-bo Feng and Gang Chen). Each participant was classified based on pathological pattern, tumor size, and clinicopathologic grade according to the IASLC 2009 criteria [18].

### RNA isolation and RT-qPCR

Total RNA was extracted from FFPE samples from the NSCLC and matching tissues by miRNeasy Kit (QIAGEN, KJVenlo, The Netherlands) according to the manual instructions. The RNA concentration was quantified using a NanoDrop 2000 instrument (Wilmington, DE, USA). Then, reverse transcription synthesis of complimentary DNA (cDNA) was conducted on First Strand cDNA Synthesis Kit (Thermo Scientific, USA), followed by PCR reaction on an Applied Biosystems PCR7900 instrument (Thermo Fisher Scientific, Waltham, USA). The thermal cycling steps started at 95 °C for 10 min, continued with totally 40 PCR cycles of 15 s at 95 °C and 60s at 60 °C, finally annealed at 72 °C for 5 s. RNU6B was utilized as the housekeeping miRNA for miR-204-5p. The primer sequences used in the TaqMan® MicroRNA Assays were as follows: RNU6B (Applied Biosystems,4,427,975–001093)-CGCAAGGAUGACACGCAAAUUCGUGAAGCGUUCCAUAUUUUU and miR-204-5p (Applied Biosystems, 4,427,975–000508)- UUCCCUUUGUCAUCCUAUGCCU. The RT-qPCR process was performed on an Applied Biosystems PCR7900 instrument using the protocol supplied by the manufacturer. The expression levels of the two miRNAs were compared using the 2 − ΔΔCt method [19]. All specimens were analyzed in triplicate.

### Data mining from TCGA

The Illumina HiSeq miRNA-sequencing data for miR-204-5p were downloaded and extracted from TCGA up to October 31, 2018. The Xena Public Data Hubs online analysis program (https://xena.ucsc.edu/public-hubs/) was used to calculate expression level of miR-204-5p and to assess the difference between 999 NSCLC and 91 normal tissues. The genes involved in NSCLC were also obtained from TCGA data and further analyzed with the EdgeR package. Genes with a false discovery rate (FDR)<0.05 were deemed differentially expressed genes (DEGs) and selected as standby members.

### Collection and management of miR-204-5p data

Genechips data related to miR-204-5p in NSCLC were sought in the GEO database (http://www.ncbi.nlm.nih.gov/geo/) up to October 31,2018.To evaluate the clinical application of miR-204-5p for NSCLC, data on documented expression of miR-204-5p between NSCLC and non-tumorous controls were collected from the following databases: PubMed, Web of Science, Wiley online library, Springerlink, Embase, Chinese National Knowledge Infrastructure, Chinese Biomedical Database, Chinese VIP and Wan Fang data resources. The data retrieval entry was as follows: (MicroRNA OR miRNA OR “Micro RNA” OR “Small Temporal RNA” OR “non-coding RNA” OR ncRNA OR “small RNA”) AND (lung OR pulmonary OR respiratory OR bronchi OR bronchioles OR alveoli OR pneumocytes OR “air way”) AND (cancer OR carcinoma OR tumor OR neoplas* OR malignan* OR adenocarcinoma).

The microarray chip data and publications had to fulfill the following conditions for inclusion in the current study:1) the research object focused on human beings, 2) the publication was in English or Chinese, 3) participants were confirmed cases with NSCLC, 4) cases included normal controls and contained at least 2 samples, and 5) pertinent expression of miR-204-5p in NSCLC and corresponding non-tumorous specimens was explored. Exclusion criteria included:1) duplicate selections of studies, conference abstracts, expert opinions, case reports, comments, letters, editorial or reviews, 2) articles with in vitro *or* in vivo experiments or human xenografts, 3) data with no information about miR-204-5p expression, and 4) publications not written in English or Chinese.

Items from the eligible datasets and reports included for further investigation were: series accession, the lead author, publication year, nationality, experimental platform, sample size, types of sample, research techniques, amount of miR-204-5p and threshold value. The above screening procedures were repeated by two veteran researchers.

### Prediction and analyses of miR-204-5p target genes

MiRwalk 2.0, an online miRNA-target search tool that integrates 12 prediction programs (miRWalk, miRanda, miRDB, MicroT4, miRMap, miRNAMap, miRBridge, PITA, PICTAR2, RNAhybrid, RNA22 and TargetScan), was applied to predict the target genes for subsequent analyses. Only genes that co-occurred in at least six databases were deemed eligible. Due to the decrease of miR-204-5p in NSCLC, the target genes were expected to be expressed at a higher level to a large extent, so up-regulated DEGs from TCGA were adopted for further work. The final estimated objects for miR-204-5p were derived from the intersection of online databases and TCGA.

The selected candidate DEGs were then processed in the Database for Annotation, Visualization, and Integrated Discovery (DAVID) v6.8 (https://david-d.ncifcrf.gov/) to obtain the gene ontology (GO) annotation as well as the Kyoto Encyclopedia of Genes and Genomes (KEGG) pathway analysis. *P* < 0.05 was regarded as the cut-off. Further information about the interaction between the proteins encoded by DEGs was obtained using the Search Tool for the Retrieval of Interacting Genes (STRING) (http://www.string-db.org/) and Cytoscape 3.6.1 to establish a protein-protein interaction (PPI) network for DEGs that participated in the top three GO items and KEGG pathways. In this study, the selection criterion for hub genes was based on the degree of connection among pitch points in the PPI network. The mRNA expression levels of the hub genes were also accessed from GEPIA (http://gepia.cancer-pku.cn), and their protein variations were validated in the Human Protein Atlas (THPA) (https://www.proteinatlas.org/).

### Transcription factor prediction

Transcription factors (TFs) that were likely to related to miRNA-204-5p and/or hub genes were predicted from public databases, followed by collection of experimentally confirmed targets from literature. Relevant TFs that were able to influence miR-204-5p were mainly predicted using three different online databases that provided estimated relationships between TFs and marker genes: Gene Transcription Regulation Database (GTRD, http://gtrd.biouml.org/), HTFtarget database (http://bioinfo.life.hust.edu.cn/hTFtarget#!/) and TransmiR v2.0 database (http://www.cuilab.cn/transmir). The TFs that modulated hub genes were acquired from GTRD and HTFtarget simultaneously. Precise information was obtained from the intersection of the predictions for combinatorial utilization. The relationships between these can be described as TF-miRNA (GTRD ∩ HTFtarget ∩ TransmiR) ∩ TF-hub genes (GTRD ∩HTFtarget). The predicted transcription factor binding sites (TFBSs) were retrieved from the JASPAR database (http://jaspar.genereg.net/), and the sequences were derived from the positive-sense strand with the highest score. Literature mining was performed with combined keywords (MicroRNA OR miRNA OR “Micro RNA” OR “Small Temporal RNA” OR “non-coding RNA” OR ncRNA OR “small RNA”) AND (transcription factor OR transcriptional factor) AND (cancer OR carcinoma OR tumor OR neoplasm* OR malignant* OR adenocarcinoma) to confirm the relationships between motifs and NSCLC or other types of cancers. Synergistic co-regulatory motifs of miR-204-5p network were constructed based on the expected regulation and literature confirmation.

### Statistical analysis

Results of miR-204-5p expression were reported as mean ± standard deviation (SD). Student’s t-test was used to compare differences in miR-204-5p expression measured by RT-qPCR or raw expression data. One-way analysis of variance (ANOVA) was conducted to evaluate the characteristics of miR-204-5p distribution among groups including three or more variates. Statistical analyses were performed using SPSS v22.0 (SPSS Inc., Chicago, IL, USA).

Data from GEO were first individually processed for acquisition of standard mean deviation (SMD) by meta-analysis, followed by their integration with TCGA and the literature to evaluate distinct expression and potential application prospects of miR-204-5p. The analytical methods in meta-analyses were identical to those used in previous studies [20, 21], and analysis was conducted using by Stata 12.0 (Stata Corp LP, College Station, USA). The role of miR-204-5p in NSCLC diagnosis was studied using receiver operating characteristic (ROC) and summarized receiver operating characteristics (SROC) curves were respectively constructed in accordance with the previous studies [22].

The RT-qPCR data were divided into low-level and high-level groups in according to the median expression level of miR-204-5p(Median = 3.75). The association between survival data in the two groups and miR-204-5p expression were analyzed by Kaplan-Meier (K-M) curves and univariate Cox regression analysis using SPSS v22.0. The hazard ratio (HR), 95% confidence interval (95% CI), and other data available from public resources were either extracted directly or obtained indirectly by recommendations of Tierney et al. [23]. Comprehensive meta-analysis was then preformed for the HRs to appraise the efficiency of miR-204-5p in NSCLC prognosis.

In this work, *P* < 0.05 was considered as statistically significant. A random effects model was considered valid when a large heterogeneity was defined with reference to I^2^ greater than 50% or P less than 0.1; otherwise a fixed- coefficient model was in usage [24–27].

## Results

### Differential expression and clinical characteristics of miR-204-5p in NSCLC

**Fig. 2:**
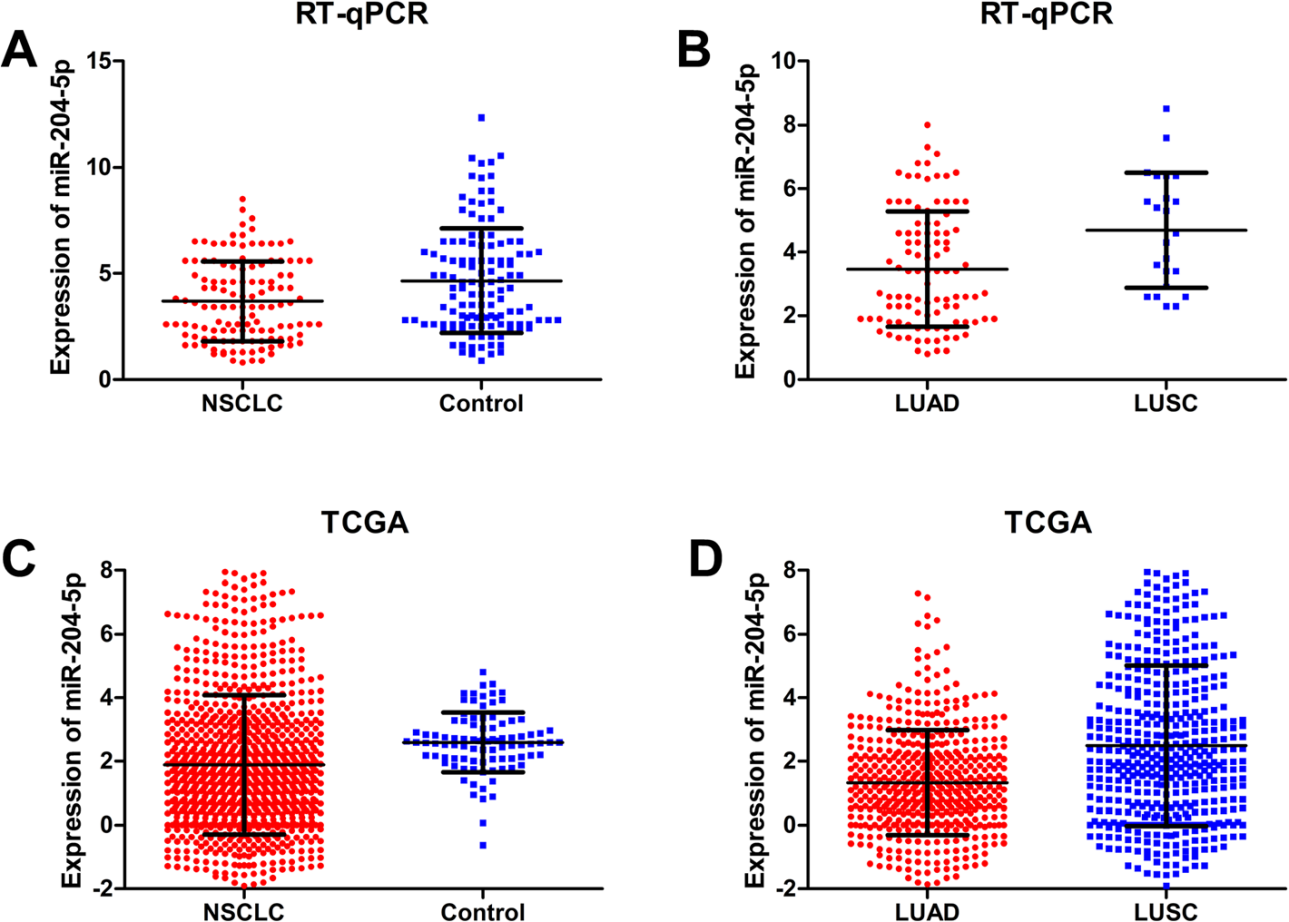
Expression of miR-204-5p in non-small cell lung cancer (NSCLC) and subgroups derived from RT-qPCR and TCGA database. **a** Scatter plot for RT-qPCR indicated significantly lower miR-204-5p expression in NSCLC tissues (3.6760 ± 1.87670) than in the controls (4.6487 ± 2.46888) (P = 0.001). **b** Expression differences for miR-204-5p between lung adenocarcinoma (LUAD) and lung squamous cell carcinoma (LUSC) determined by RT-qPCR. The decrease was more obvious in LUAD (3.4663 ± 1.82397) than in LUSC (4.6870 ± 1.80638) (P = 0.006). **c** Expression level of miRNA-sequencing data from TCGA revealed significantly lower miR-204-5p expression in NSCLC tissues (1.8877 ± 2.18763) than in healthy control tissues (2.5944 ± 0.9404) (P = 0.000). **d** Expression comparison of miR-204-5p between LUAD (1.3331 ± 1.64315) and LUSC from TCGA (2.4922 ± 2.52336). The difference was consistent with the foregoing data of RT-qPCR. (P = 0.000)

Relative quantitative expression and the fundamental characteristics of miR-204-5p in the research subjects are listed in Table 1. The RT-qPCR results (Table 1 and Fig. 2a) showed a statistically significant difference in the quantitative variation of miR-204-5p between NSCLC and normal adjacent tissues (*P* = 0.001). Statistical differences were also found for gender, tumor size, histological type, vascular invasion, tumor node metastasis (TNM) grade, and lymph node metastasis (P<0.05). Differences of miR-204-5p in expression between pathological types were assessed by analyzing RT-qPCR data grouped into lung adenocarcinoma (LUAD) and lung squamous cell carcinoma (LUSC) (Table 2, Table 3 and Fig. 2b). Apart from age and smoking behavior, lower of miR-204-5p expression was noted in sex, tumor size, vascular invasion, TNM grade, lymph node metastasis, and pathological grading in the LUAD group than in the LUSC group (P<0.05). Therefore, miR-204-5p expression was reduced in NSCLC and was related to clinical parameters other than age and smoking, especially in the LUAD group.

**Table 1:** Clinicopathological parameters and the expression of miR-204-5p in NSCLC. Annotation: *n* number, *SD* standard deviation, *NSCLC* non-small cell lung cancer. a, paired sample’s t test performed to compare miR-204-5p expression between NSCLC and the controls; Independent sample’s t test processed to assess relationships between miR-30d-5p expression and the clinicopathological parameters of NSCLC. TNM, tumor, node, metastasis; b, One-way ANOVA preformed to evaluate distributive feature of miR-204-5p in three or more groups of clinicopathological parameters

Clinicopathological parameters		n	Relevant expression of miR-204-5p (2^−ΔCq^)		
			Mean ± SD	t/F-value	*p*-value
Tissue	NSCLC	125	3.6760 ± 1.87670	-3.507^a^	0.001
	Non-cancer	125	4.6487 ± 2.46888		
Gender	Male	75	4.0067 ± 1.91843	2.461	0.015
	Female	50	3.1800 ± 1.71357		
Age (years)	< 60	57	3.9526 ± 1.81847	1.517	0.132
	> = 60	68	3.4441 ± 1.90650		
Smoke	No	38	4.3368 ± 1.70205	−0.108	0.914
	Yes	30	4.3833 ± 1.83041		
Histological type	Adenocarcinoma	101	3.4663 ± 1.82397	−2.902	0.004
	Squamous carcinoma	23	4.6870 ± 1.80638		
Tumor size	<=3 cm	60	3.2417 ± 1.78547	−2.540	0.012
	> 3 cm	65	4.0769 ± 1.88280		
Vascular invasion	No	90	4.2233 ± 1.68876	5.898	< 0.001
	Yes	35	2.2686 ± 1.59609		
TNM	I-II	54	4.0870 ± 1.96383	2.167	0.032
	III-IV	71	3.3634 ± 1.75770		
Lymph node metastasis	No	56	4.2089 ± 1.95897	2.948	0.004
	Yes	69	3.2435 ± 1.70142		
Pathological grading	I	17	4.2176 ± 1.94140	2.797^b^	0.065
	II	78	3.8090 ± 1.85404		
	III	30	3.6760 ± 1.87670

**Table 2:** Clinicopathological parameters and the expression of miR-204-5p in LUAD. Annotation: LUAD, lung adenocarcinoma. a, paired sample’s t test performed to compare miR-204-5p expression between NSCLC and the controls; Independent sample’s t test processed to assess relationships between miR-30d-5p expression and the clinicopathological parameters of NSCLC. TNM, tumor, node, metastasis; b, One-way ANOVA preformed to evaluate distributive feature of miR-204-5p in three or more groups of clinicopathological parameters

Clinicopathological parameters		n	Relevant expression of miR-204-5p (2^−ΔCq^)		
			Mean ± SD	t/F-value	p-value
Tissue	LUAD	101	3.4663 ± 1.82397	-2.731^a^	0.007
	Non-cancer	101	4.2786 ± 2.36824		
Gender	Male	56	3.7768 ± 1.91937	1.934	0.056
	Female	45	3.0800 ± 1.63729		
Age (years)	< 60	41	3.7390 ± 1.85039	1.245	0.216
	> = 60	60	3.2800 ± 1.79734		
Smoke	No	26	4.1000 ± 1.67141	−0.695	0.491
	Yes	18	4.4611 ± 1.72768		
Tumor size	<=3 cm	53	3.0906 ± 1.72362	−2.218	0.029
	> 3 cm	48	3.8813 ± 1.85915		
Vascular invasion	No	70	4.1114 ± 1.63215	6.286	< 0.001
	Yes	31	2.0097 ± 1.34123		
TNM	I-II	44	3.8864 ± 1.87190	2.066	0.041
	III-IV	57	3.1421 ± 1.73339		
Lymph node metastasis	No	45	4.0556 ± 1.86822	3.027	0.003
	Yes	56	2.9929 ± 1.65660		
Pathological grading	I	17	4.2176 ± 1.94140	5.477^b^	0.006
	II	61	3.6279 ± 1.81752		
	III	23	2.4826 ± 1.36070

**Table 3:** Clinicopathological parameters and the expression of miR − 204-5p in LUSC. Annotation: LUSC, lung squamous cell carcinoma. The rest were the same as Table 1. a, paired sample’s t test performed to compare miR-204-5p expression between NSCLC and the controls; Independent sample’s t test processed to assess relationships between miR-30d-5p expression and the clinicopathological parameters of NSCLC. TNM, tumor, node, metastasis; b, One-way ANOVA preformed to evaluate distributive feature of miR-204-5p in three or more groups of clinicopathological parameters

Clinicopathological parameters		n	Relevant expression of miR-204-5p (2^−ΔCq^)		
			Mean ± SD	t/F-value	p-value
Tissue	LUSC	23	4.6870 ± 1.80638	-2.264^a^	0.029
	Non-cancer	23	6.0217 ± 2.17547		
Gender	Male	18	4.8556 ± 1.68041	0.844	0.408
	Female	5	4.0800 ± 2.31452		
Age (years)	< 60	15	4.6933 ± 1.52572	0.020	0.985
	> = 60	8	4.6750 ± 2.36628		
Smoke	No	12	4.8500 ± 1.72495	0.444	0.662
	Yes	11	4.5091 ± 1.95931		
Tumor size	<=3 cm	7	4.3857 ± 1.96759	−0.520	0.608
	> 3 cm	16	4.8188 ± 1.78222		
Vascular invasion	No	20	4.6150 ± 1.86471	−0.485	0.633
	Yes	3	5.1667 ± 1.56950		
TNM	I-II	10	4.9700 ± 2.21512	0.650	0.523
	III-IV	13	4.4692 ± 1.47783		
Lymph node metastasis	No	11	4.8364 ± 2.28266	0.364	0.721
	Yes	12	4.5500 ± 1.32150		
Pathological grading	I	0		0.038^b^	0.848
	II	16	4.6375 ± 1.80032		
	III	7	4.8000 ± 1.95959		

### Verification of miR-204-5p expression in TCGA

In this validation set, the levels of miR-204-5p were markedly decreased in NSCLC when compared to the normal control tissues (*P* = 0.000) (Fig. 2c). The TCGA Records were divided into TCGA-LUAD (containing 521 tumor cases and 46 controls) and TCGA-LUSC (478 tumor cases and 45 controls) due to the possibility of expression differences. The detected levels of miR-204-5p was lower in TCGA-LUAD, which was consistent with our results (*P* = 0.006) (Fig. 2d).

### Results of data mining

**Fig. 3:**
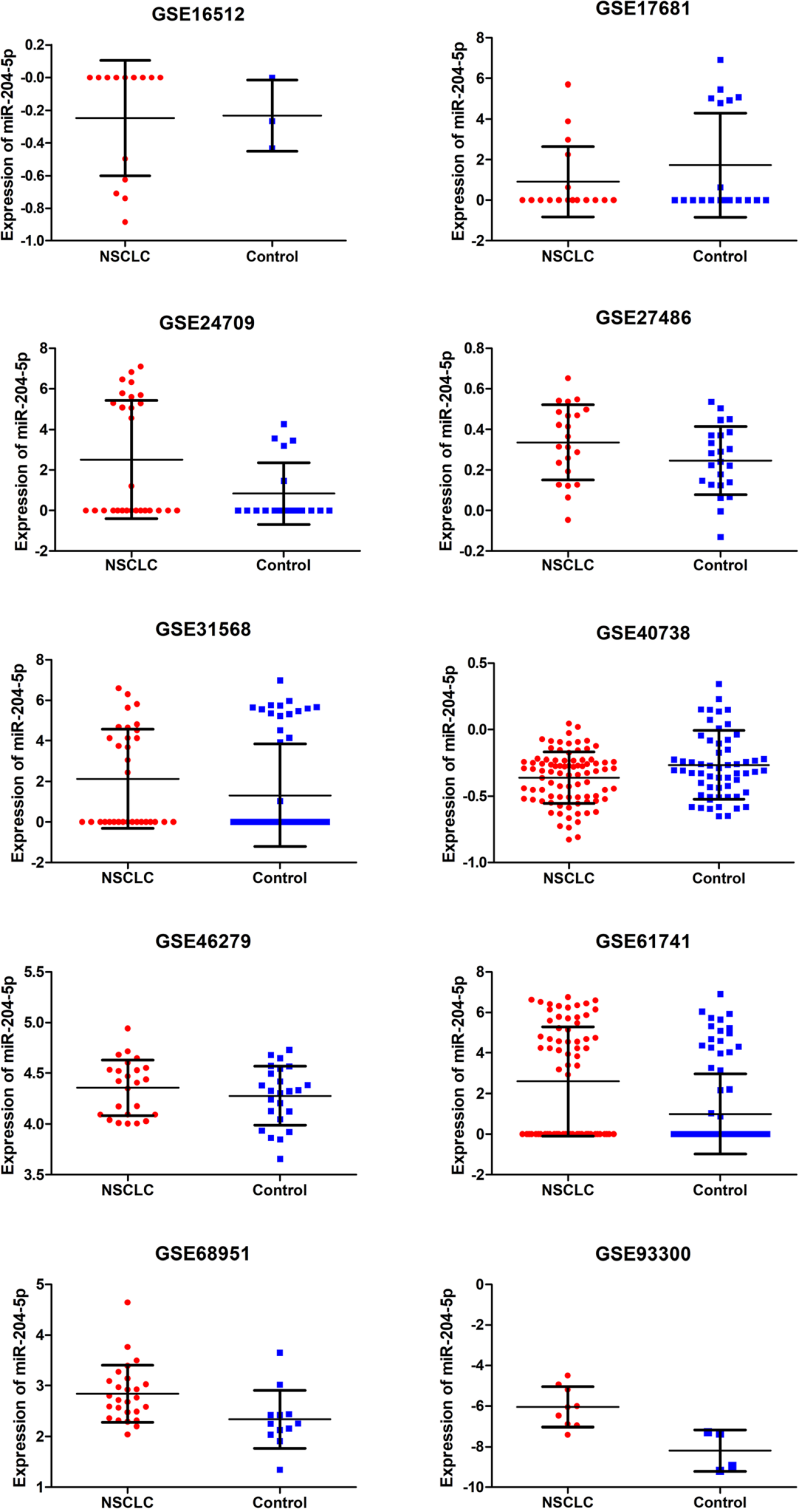
Scatter point plots for miR-204-5p expression in plasma from GSE datasets

**Fig. 4:**
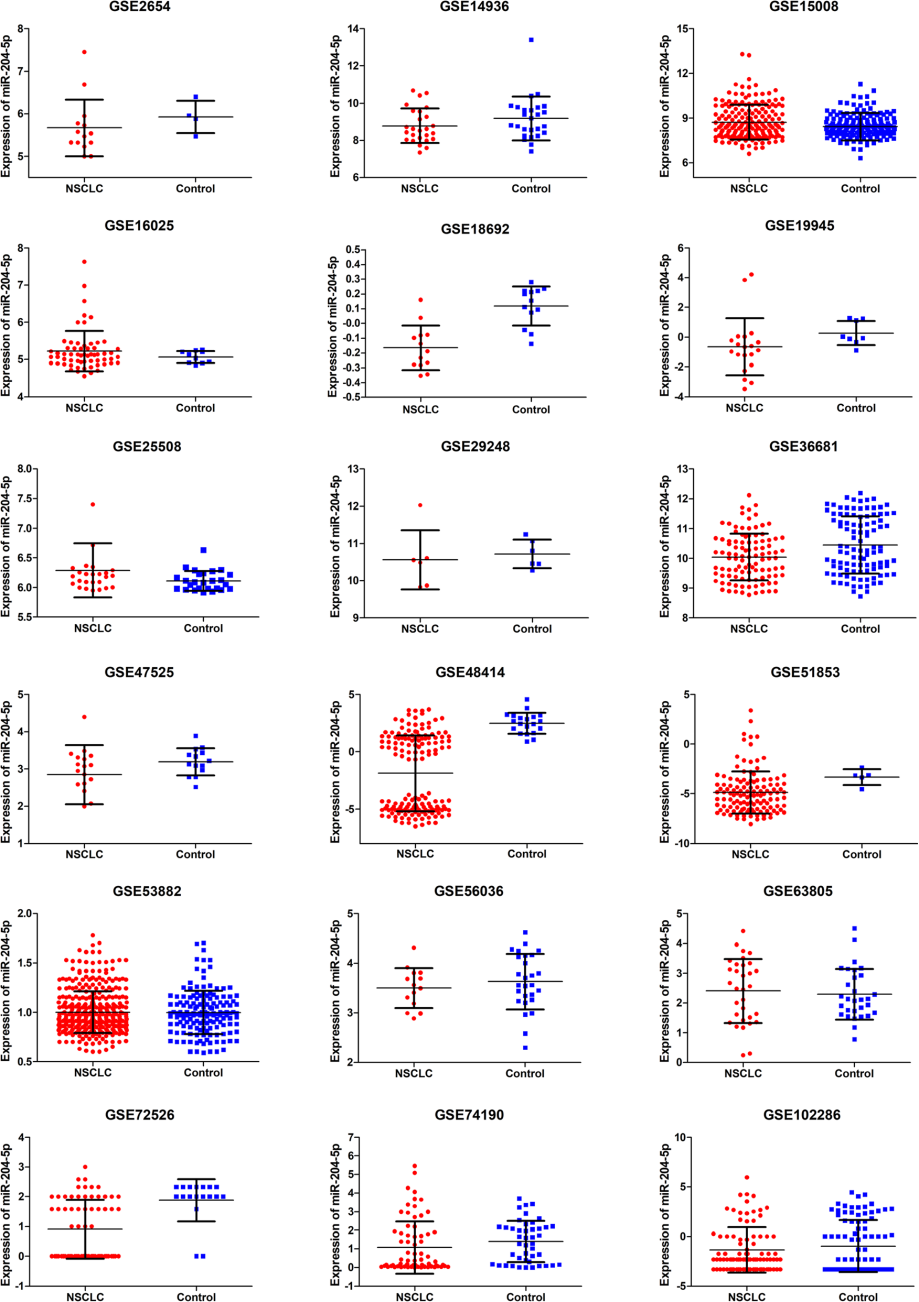
Scatter point plots for miR-204-5p expression in tissues from GSE datasets

Another 33 findings were selected for further analyses: 28 GEO datasets, 1 TCGA and 4 qualified publications. The first 11 investigations were involved monitoring of plasma samples, whereas the 22 analyzed solid tissues. The study by Guo W [15] was the only one derived from PubMed in this portion; papers 1 through 3 [28–30] were Chinese articles. The included datasets contained 3168 NSCLC cases and 1542 control samples. After acquisition of miRNA-204-5p from GEO, the means and SDs were calculated to assess its status in NSCLC. Detailed outcomes are listed in Table 4 and scatter point plots are presented in Fig. 3, 4.

**Table 4:** Detailed information of all datasets used in SMD metaanalysis: eligible GEO datasets, TCGA, qualified publications and our RT-qPCR (represented as Current study). P<0.05 was considered as significant. Annotation: *SMD*standard mean deviation, *NO*number, *RTqPCR*realtime quantitative polymerase chain reaction. Since no citations were reflected for GSE24709, GSE46729, GSE93300, GSE19945 and GSE74190, websites were the alternatives

ID	Lead author	Year	Country	Source	Platform	Experimental type	Citation	Cancer No.	Control No.	T value	*P*value
GSE16512	Lodes MJ	2009	USA	plasma	GPL8686	array	[31]	3	14	0.066	0.057
GSE17681	Keller A	2009	Germany	plasma	GPL9040	array	[32]	17	19	−1.104	0.009
GSE24709	Keller A	2011	Germany	plasma	GPL9040	array	[33]	28	19	2.289	0.000
GSE27486	Patnaik SK	2010	USA	plasma	GPL11432	array	[34]	22	23	1.699	0.518
GSE31568	Keller A	2011	Germany	plasma	GPL9040	array	[35]	32	70	1.527	0.363
GSE40738	Patnaik SK	2012	USA	plasma	GPL16016	array	[36]	86	59	−2.561	0.125
GSE46729	Godrey A	2014	USA	plasma	GPL8786	array	[37]	24	24	0.955	0.945
GSE61741	Keller A	2014	Germany	plasma	GPL9040	array	[38]	73	94	4.427	0.000
GSE68951	Leidinger P	2015	Germany	plasma	GPL16770	array	[39]	26	12	2.553	0.773
PMID:26497897	Guo W	2015	China	plasma	NR	RT-qPCR	[15]	126	50	NR	< 0.001
GSE93300	Liu X	2017	China	plasma	GPL21576	array	[40]	9	4	3.557	0.748
GSE2564	Lu J	2005	USA	tissue	GPL1987	array	[41]	14	4	−0.731	0.396
GSE14936	Seike M	2009	USA	tissue	GPL8879	array	[42]	26	26	−1.344	0.654
GSE15008	Tan X	2009	China	tissue	GPL8176	array	[43]	187	174	2.883	0.000
GSE16025	Raponi M	2009	USA	tissue	GPL5106	array	[44]	61	10	0.916	0.111
GSE18692	Puissegur M	2009	France	tissue	GPL4718	array	[45]	13	13	−5.072	0.617
GSE19945	Ohba T	2010	Japan	tissue	GPL9948	array	[46]	20	8	−1.305	0.289
GSE25508	Guled M	2011	Finland	tissue	GPL7731	array	[47]	26	26	1.868	0.080
GSE29248	Ma L	2010	China	tissue	GPL8179	array	[48]	6	6	−0.431	0.474
GSE36681	Jang JS	2012	USA	tissue	GPL8179	array	[49]	103	103	−3.282	0.001
GSE47525	van Jaarsveld MT	2013	Netherlands	tissue	GPL17222	array	[50]	18	14	−1.499	0.103
GSE48414	Bjaanaes MM	2014	Norway	tissue	GPL16770	array	[51]	154	20	−5.891	0.000
GSE51853	Arima C	2014	Japan	tissue	GPL7341	array	[52]	126	5	−1.63	0.103
GSE53882	Pu HY	2014	China	tissue	GPL18130	array	[53]	397	151	0.148	0.933
GSE56036	Fujita Y	2014	Japan	tissue	GPL15446	array	[54]	14	27	−0.756	0.204
GSE63805	Robles AI	2014	USA	tissue	GPL18410	array	[55]	32	30	0.449	0.074
GSE72526	Gasparini P	2015	Switzerland	tissue	GPL20275	array	[56]	67	18	− 3.904	0.000
GSE74190	Jin Y	2015	China	tissue	GPL19622	array	[57]	72	44	−1.306	0.141
GSE102286	Mitchell KA	2017	USA	tissue	GPL23871	array	[58]	91	88	−1.087	0.003
TCGA	NR	NR	NR	tissue	NR	array	NR	999	91	−3.055	0.000
Literature 1	Li LX	2017	China	tissue	NR	RT-qPCR	[28]	39	39	NR	<0.01
Literature 2	Xu YZ	2018	China	tissue	NR	RT-qPCR	[30]	60	60	9.361	0.000
Literature 3	Wang QC	2018	China	tissue	NR	RT-qPCR	[29]	72	72	11.028	<0.01
Current study	NR	NR	China	tissue	NR	RT-qPCR	NR	125	125	−3.507	0.007

Four GEO datasets assayed in plasma samples showed significant differences, but only two (GSE17681 and PMID:26497897) demonstrated the decreased level of miRNA-204-5p expression in NSCLC. Another 8 investigations performed in tissues were reflected in statistical significance except for the TCGA results; seven of these indicated a downregulation of miRNA-204-5p expression in NSCLC tissue specimens.

### Integrated meta-analyses of miR-204-5p datasets in NSCLC

**Fig. 5:**
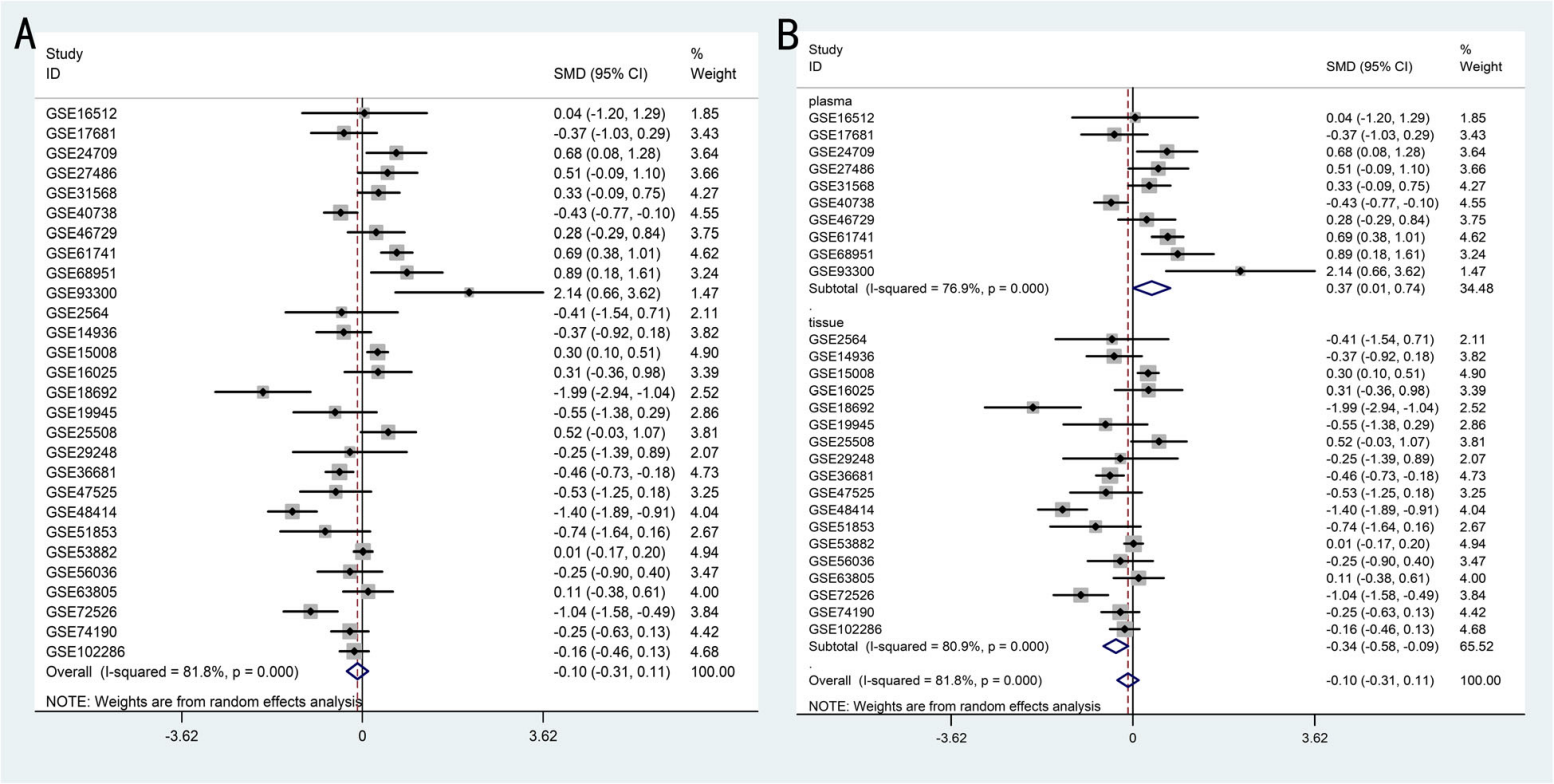
Forest plot and subgroup analysis of miR-204-5p expression levels based on GEO data. A: Non-small cell lung cancer (NSCLC) vs. healthy controls; random-effects model. B: Subgroup analysis layered by specimen source, plasma and tissue; random-effects model

Each meta-analysis was first individually processed to evaluate level of miR-204-5p in the GEO data which covered 1747 NSCLC patients and 1105 control samples. Dysregulation of miR-204-5p was evident in NSCLC (SMD = − 0.098, 95% CI: − 0.310 to 0.114), but with poor statistical significance (*P* = 0.366) and high heterogeneity (I^2^ = 81.8%, *P* = 0.000) (Fig. 5a). Unexpected outcomes were obtained from subgroup meta-analysis, which indicated that the decrease was significantly different in both plasma (SMD = 0.374, 95% CI: 0.005 to 0.743, *P* = 0.047) and (SMD = − 0.098, 95% CI: − 0.310 to 0.114, *P* = 0.007) tissues, but also suggested a more sensitive response in cancerous tissues and evident heterogeneity (I^2^ > 90%, P = 0.000) as well (Fig. 5b). Random models were used to reduce the impact of heterogeneity.

**Fig. 6:**
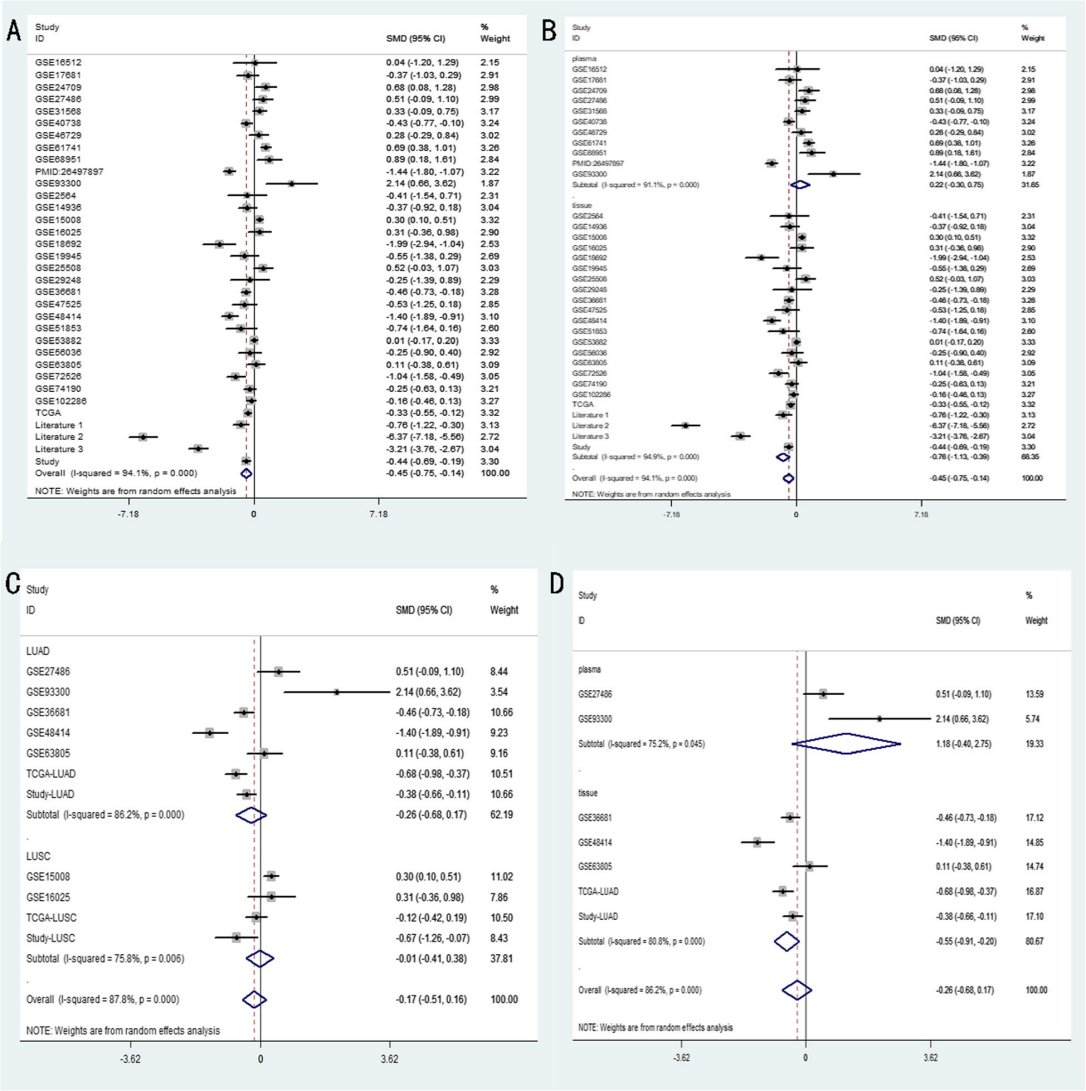
Forest plot and subgroup analysis of miR-204-5p level in non-small cell lung cancer (NSCLC)**. a** NSCLC vs. healthy controls; random-effects model. **b** Subgroup analysis layered by specimen source, plasma and tissue; random-effects model. **c** Subgroup analysis between lung adenocarcinoma (LUAD) and lung squamous cell carcinoma (LUSC); random-effects model. **d** Subgroup analysis of LUAD sources, plasma and tissue; random-effects model

An integrative meta-analysis of the entire data collection obtained from GEO, TCGA, publications and our RT-qPCR analyses was conducted to obtain a more precise assessment of miR-204-5p expression. Down-regulation of miR-204-5p in NSCLC (overall pooled SMD = − 0.447, 95% CI: − 0.750 ~ − 0.144, *P* = 0.004) was confirmed by the forest graph displayed in Fig. 6a, and the reduction was more significant in tissues (SMD = − 0.760, 95% CI: − 1.132 to − 0.378, P = 0.000) than in plasma (SMD = 0.224, 95% CI: − 0.301 to 0.749, *P* = 0.403) (Fig. 6b). Since substantial heterogeneity (I^2^ > 90%, P = 0.000) between data sources was noted between the data sources, a random model was adopted. The decline in miR-204-5p expression was more distinct in LUAD (SMD = − 0.258, 95% CI: − 0.685 to 0.169) than LUSC (SMD = − 0.012, 95% CI: − 0.406 to 0.382), though this subgroup analysis displayed weak statistical significance (*P* = 0.313) and considerable heterogeneity (I^2^ = 87.8%) (Fig. 6c). Furthermore, the reduction in miR-204-5p expression seemed more evident in LUAD tissues (SMD = − 0.554, 95% CI:-0.909 to − 0.199) than in plasma (SMD =1.176, 95% CI: − 0.397 to 2.748), but again the differences were not statistically significant (*P* = 0.236) and the data showed marked heterogeneity (I^2^ = 86.2%)(Fig. 6d).

### Clinical role of miR-204-5p in NSCLC

**Fig. 7:**
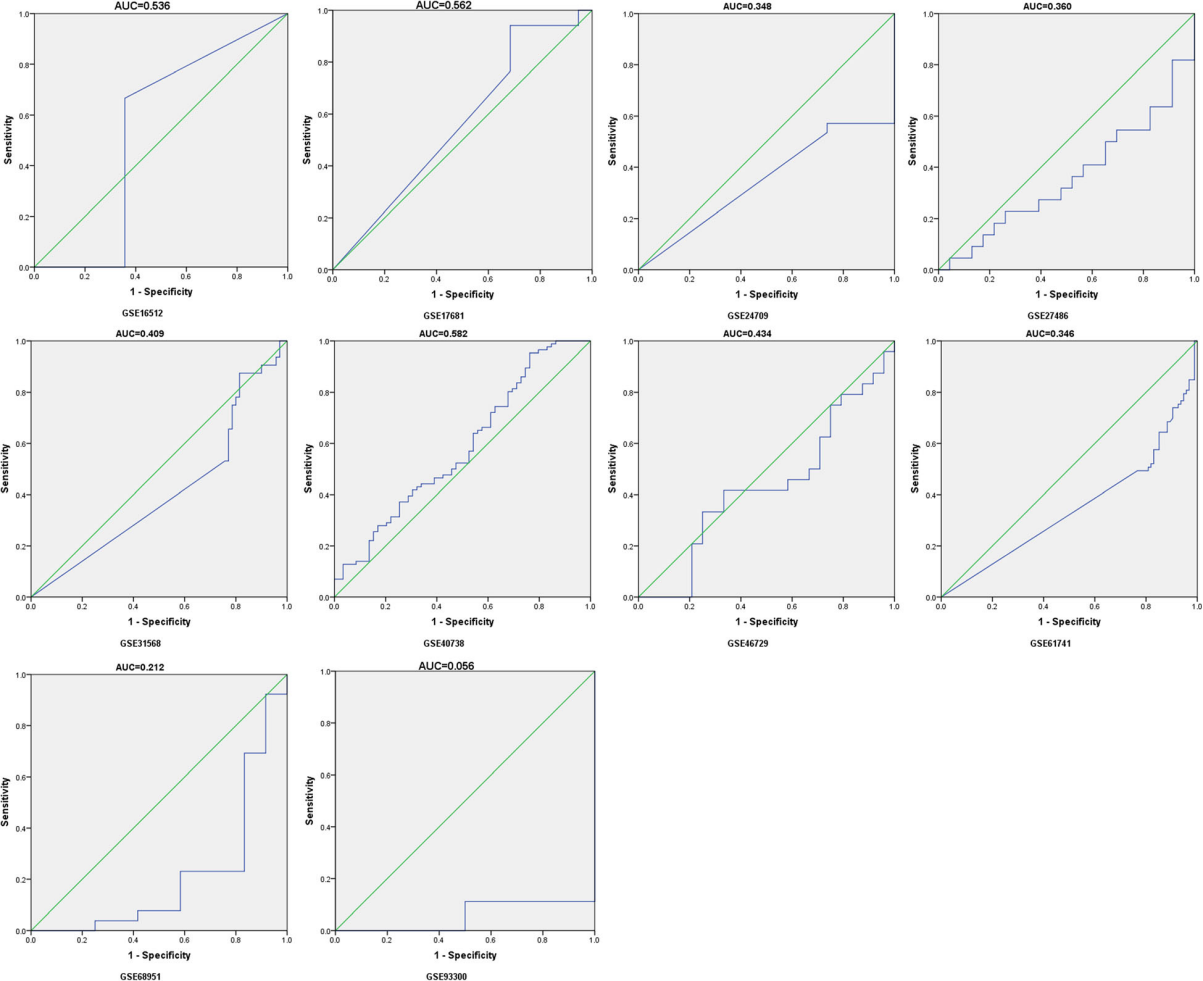
ROC curves of miR-204-5p in non-small cell lung cancer (NSCLC) plasma

**Fig. 8:**
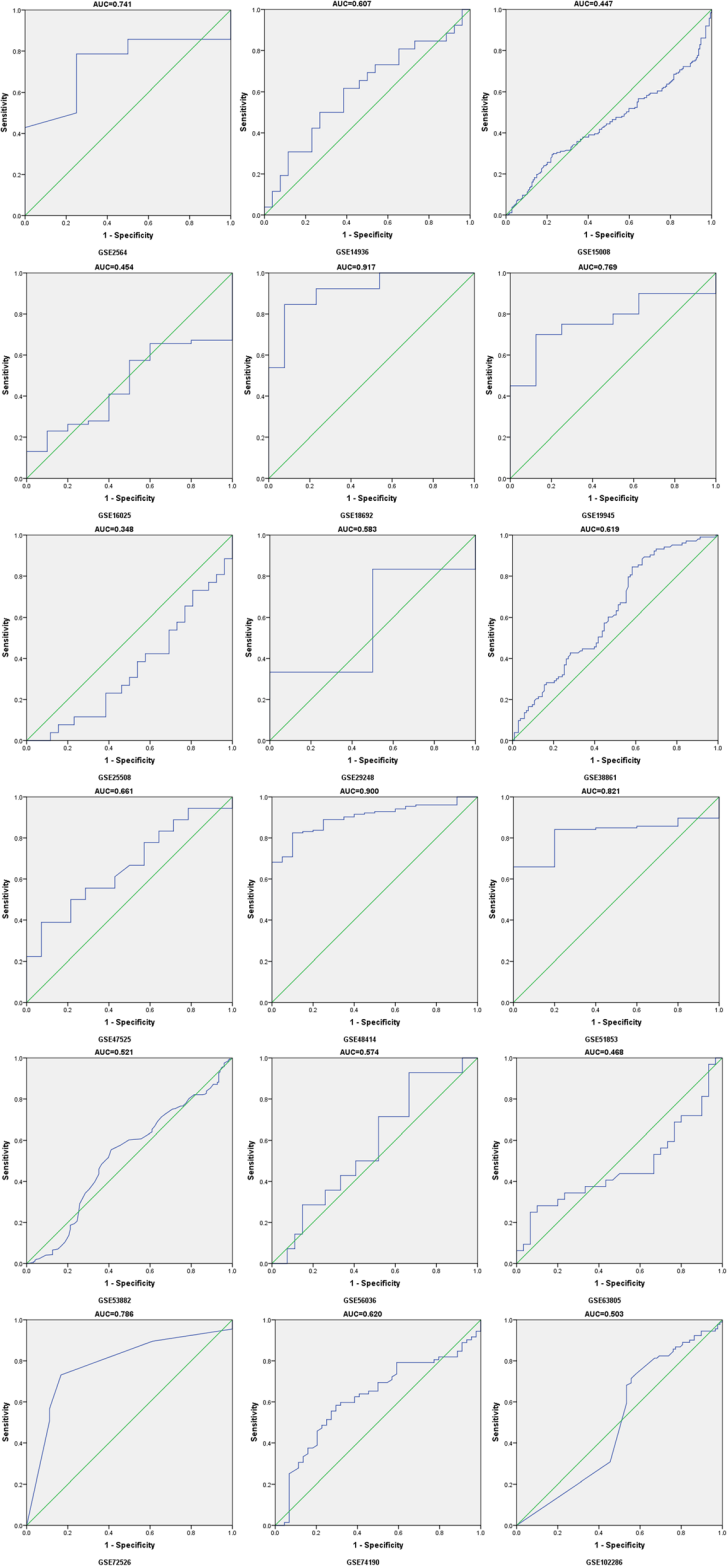
ROC curves of miR-204-5p in non-small cell lung cancer (NSCLC) tissues

**Fig. 9:**
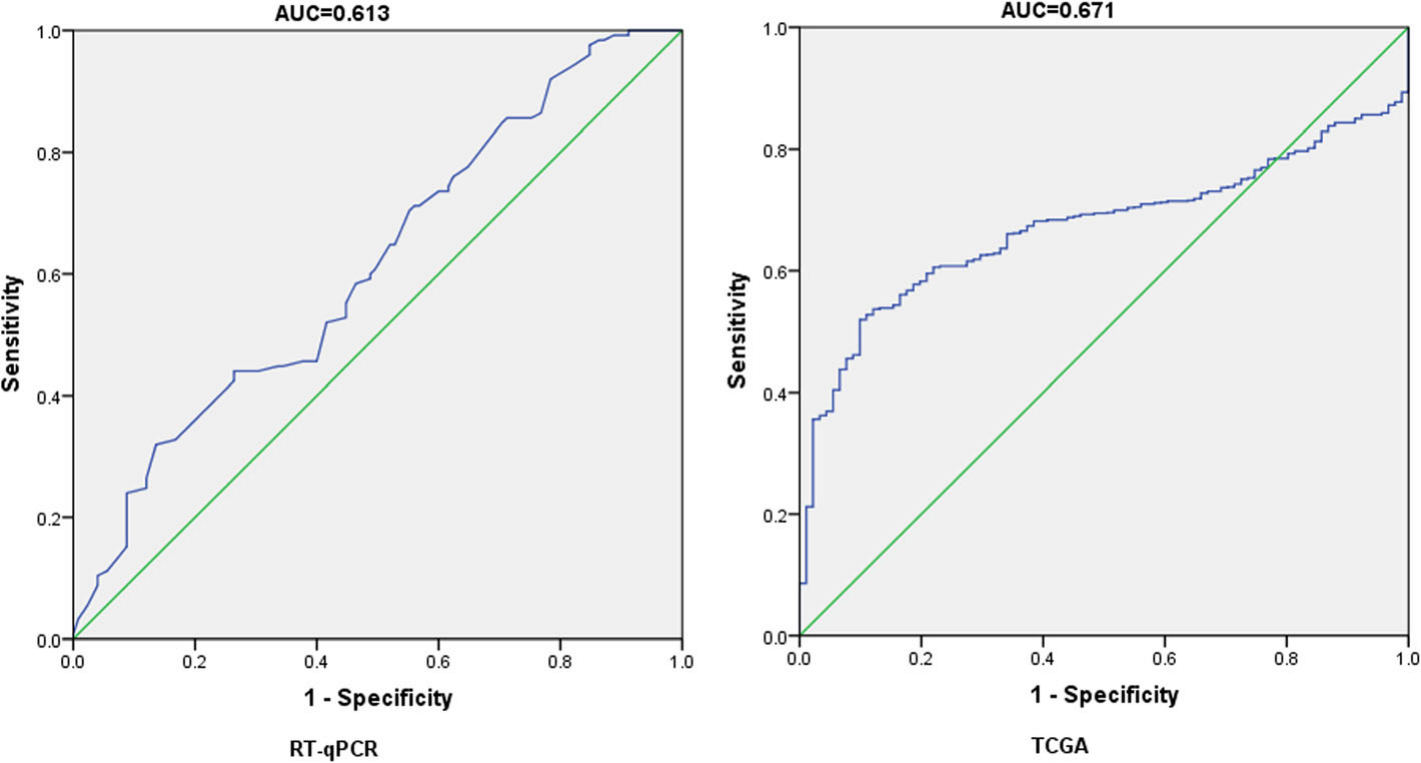
ROC curves for RT-qPCR and TCGA data

In total,31 records, which included 4368 samples derived from 28 GEO datasets, 1 TCGA, 1 publication and our study (Table 5), were used for diagnosis meta-analysis to survey the clinical role of miR-204-5p in NSCLC. Prior to the diagnosis meta-analysis, ROC curve for every case was generated and 4-fold table data were calculated. As showed in Figs. 7 and 8, the ROC curves presented varied diagnostic value with most of them revealing relatively high region in solid tissues, in agreement with TCGA and our study (Fig. 9).

**Table 5:** Information and ROC fourfold table for all datasets. Annotation: No, number of NSCLC cases and the matched group, respectively; AUC, area under the receiver operating characteristic curve; *TP*true positive, *FN*false negative, *FP*false positive, *TN*true negative. Since no citations were reflected for GSE16512, GSE17681, GSE24709, GSE46729, GSE93300, GSE19945 and GSE74190, websites were the alternatives

ID	Author	Year	Country	Source	Citation	Cases/Controls No.	AUC	Threshold	Sensitivity	Specificity	TP	FP	FN	TN
GSE16512	Lodes MJ	2009	USA	plasma	[31]	3/14	0.536	−0.133	0.667	0.643	2	5	1	9
GSE17681	Keller A	2009	Germany	plasma	[32]	17/19	0.562	4.346	0.941	0.316	16	13	1	6
GSE24709	Keller A	2011	Germany	plasma	[33]	28/19	0.348	6.960	0.964	0.000	27	19	1	0
GSE27486	Patnaik SK	2010	USA	plasma	[34]	22/23	0.360	−0.025	0.045	0.957	1	1	21	22
GSE31568	Keller A	2011	Germany	plasma	[35]	32/70	0.409	5.016	0.875	0.816	28	57	4	13
GSE40738	Patnaik SK	2012	USA	plasma	[36]	86/59	0.582	−0.085	0.953	0.237	82	45	4	14
GSE46729	Godrey A	2014	USA	plasma	[37]	24/24	0.434	4.193	0.417	0.667	10	8	14	16
GSE61741	Keller A	2014	Germany	plasma	[38]	73/94	0.346	6.828	1.000	0.011	73	93	0	1
GSE68951	Leidinger P	2015	Germany	plasma	[39]	26/12	0.212	3.575	0.923	0.083	24	11	2	1
PMID:26497897	Guo W	2015	China	plasma	[15]	126/50	0.809	0.023	0.760	0.820	96	9	30	41
GSE93300	Liu X	2017	China	plasma	[40]	9/4	0.056	−3.499	1.000	0.000	9	4	0	0
GSE2564	Lu J	2005	USA	tissue	[41]	14/4	0.741	5.835	0.786	0.750	11	1	3	3
GSE14936	Seike M	2009	USA	tissue	[42]	26/26	0.607	8.545	0.500	0.731	13	7	13	19
GSE15008	Tan X	2009	China	tissue	[43]	187/174	0.447	7.941	0.294	0.776	55	39	132	135
GSE16025	Raponi M	2009	USA	tissue	[44]	61/10	0.454	4.813	0.131	1.000	8	0	53	10
GSE18692	Puissegur M	2009	France	tissue	[45]	13/13	0.917	−0.076	0.846	0.923	11	1	2	12
GSE19945	Ohba T	2010	Japan	tissue	[46]	20/8	0.769	−0.342	0.700	0.875	14	1	6	7
GSE25508	Guled M	2011	Finland	tissue	[47]	26/26	0.348	9.004	1.000	0.000	26	26	0	0
GSE29248	Ma L	2010	China	tissue	[48]	6/6	0.583	10.704	0.833	0.500	5	3	1	3
GSE36681	Jang JS	2012	USA	tissue	[49]	103/103	0.619	10.847	0.845	0.417	87	60	16	43
GSE47525	van Jaarsveld MT	2013	Netherlands	tissue	[50]	18/14	0.661	2.755	0.389	0.929	7	1	11	13
GSE48414	Bjaanaes MM	2014	Norway	tissue	[51]	154/20	0.900	1.503	0.825	0.900	127	2	27	18
GSE51853	Arima C	2014	Japan	tissue	[52]	126/5	0.821	−4.558	0.659	1.000	83	0	43	5
GSE53882	Pu HY	2014	China	tissue	[53]	397/151	0.521	0.965	0.554	0.589	220	62	177	89
GSE56036	Fujita Y	2014	Japan	tissue	[54]	14/27	0.574	3.960	0.929	0.333	13	18	1	9
GSE63805	Robles AI	2014	USA	tissue	[55]	32/30	0.468	1.443	0.250	0.933	8	2	24	28
GSE72526	Gasparini P	2015	Switzerland	tissue	[56]	67/18	0.786	1.793	0.731	0.833	49	3	18	15
GSE74190	Jin Y	2015	China	tissue	[57]	72/44	0.620	0.472	0.583	0.705	42	13	30	31
GSE102286	Mitchell KA	2017	USA	tissue	[58]	91/88	0.503	−0.529	0.714	0.443	65	49	26	39
TCGA	NR	NR	NR	tissue	NR	999/91	0.671	1.657	0.520	0.901	519	9	480	82
Current study	NR	NR	China	tissue	NR	125/125	0.613	2.350	0.320	0.864	40	17	85	108

**Fig. 10:**
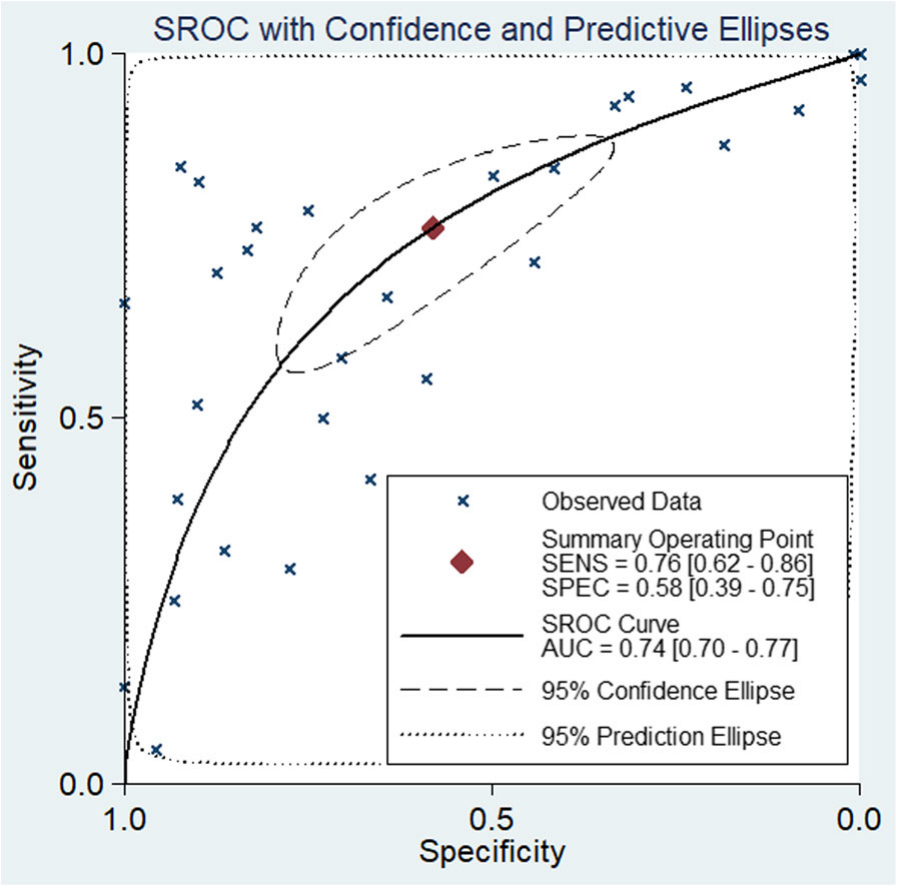
SROC curve of combinative meta-analysis data assessing the diagnostic significance of miR-204-5p in non-small cell lung cancer (NSCLC)

**Fig. 11:**
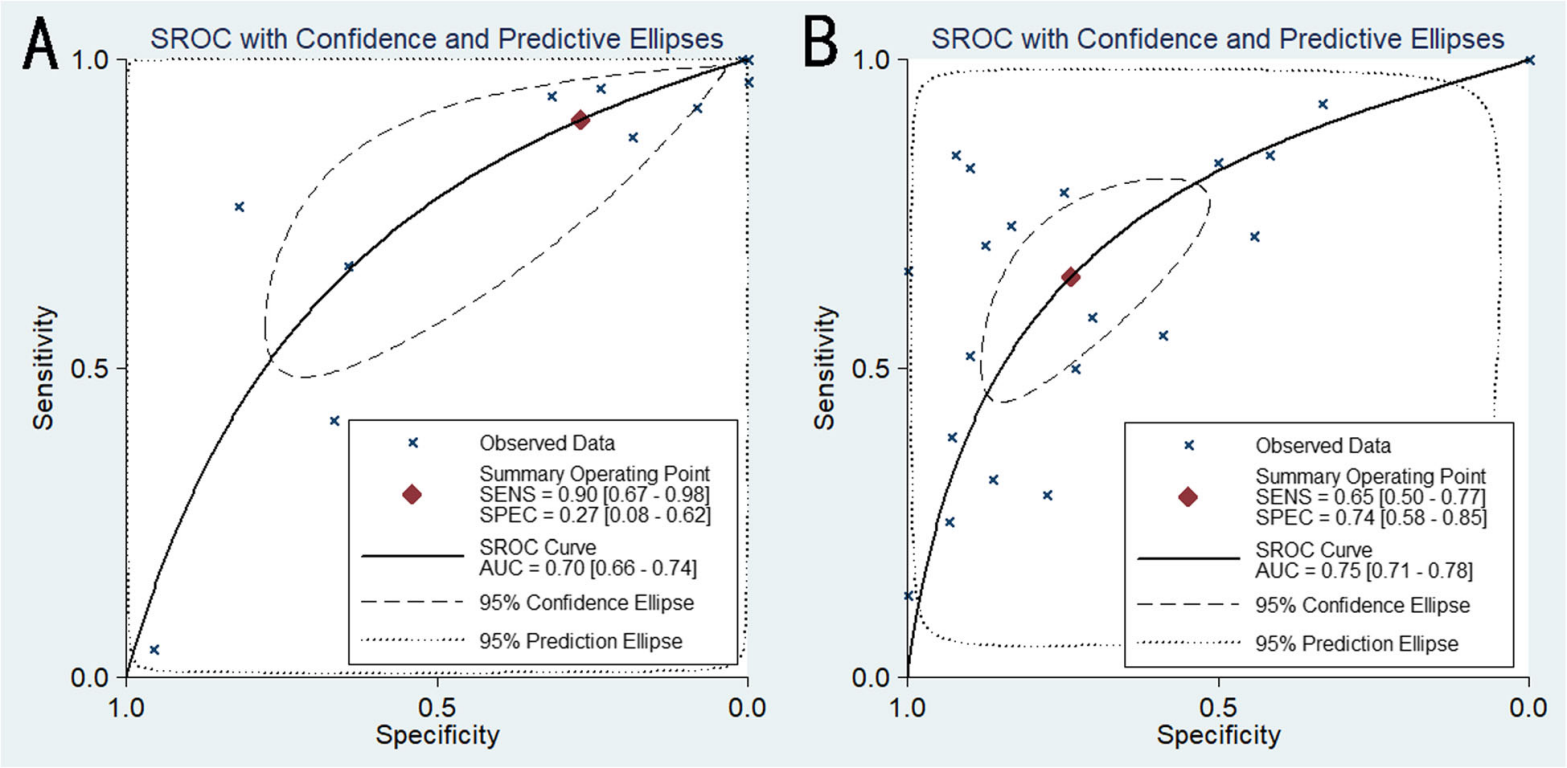
SROC curves of subgroup meta-analysis assessing the diagnostic significance of miR-204-5p in plasma and tissue. **a** Samples from plasma. **b** Samples from tissues

The miR-204-5p diagnostic accuracy and its significance in NSCLC was further examined in SROC plots integrating all GEO datasets, TCGA, publications and our study to arrive at a reliable conclusion. Simultaneous subgroup analysis was conducted on the experimental sources and tumor types. The whole combined area under the curve (AUC) was 0.74 (95% CI: 0.70–0.77) with a sensitivity and specificity of 0.76 and 0.58 respectively (Fig. 10). The AUCs from different sample origins were similar to the combined AUC, whereas polarization of the sensitivity and specificity was evident in the plasma portion (Fig. 11). The AUC was larger for the entire LUAD group (0.78, 95% CI: 0.74–0.81) than for the entire LUSC group (0.66, 95% CI: 0.62–0.70), and showed higher sensitivity (0.63 to 0.32) and lower specificity (0.78 to 0.90). However, significant heterogeneity was evident by the large Q and I^2^ values, except in the LUSC subgroup (Table 6).

**Table 6:** Diagnostic accuracy evaluation of miR-204-5p by ROC analysis. Annotation: AUC, area under the receiver operating characteristic curve; 95% CI, 95% confidence interval; LL, lower limit; UL, upper limit; Q, heterogeneity Q test; P_het_, P value of heterogeneity. LUAD, lung adenocarcinoma; LUSC, lung squamous cell carcinoma

Sample type	Study number	Enrolled number	AUC	Overall estimate			Heterogeneity			Pretest probability
				95% CI (LL-UL)	sensitivity	specificity	Q	I^2^ (%)	P_het_	
Overall	31	4368	0.74	0.70–0.77	0.76	0.58	864.488	99.77	0.000	0.686
Tissue	20	3534	0.75	0.71–0.78	0.65	0.74	328.601	99.39	0.000	0.722
Plasma	11	834	0.70	0.66–0.74	0.90	0.27	279.536	99.28	0.000	0.535
LUAD	7	1269	0.78	0.74–0.81	0.63	0.78	150.286	98.67	0.000	0.742
LUSC	4	1001	0.66	0.62–0.70	0.32	0.90	3.112	35.72	0.106	0.748
LUAD-tissue	5	1211	0.79	0.75–0.82	0.61	0.81	106.761	98.13	0.000	0.752

### Prognostic evaluation of miR-204-5p in NSCLC

**Fig. 12:**
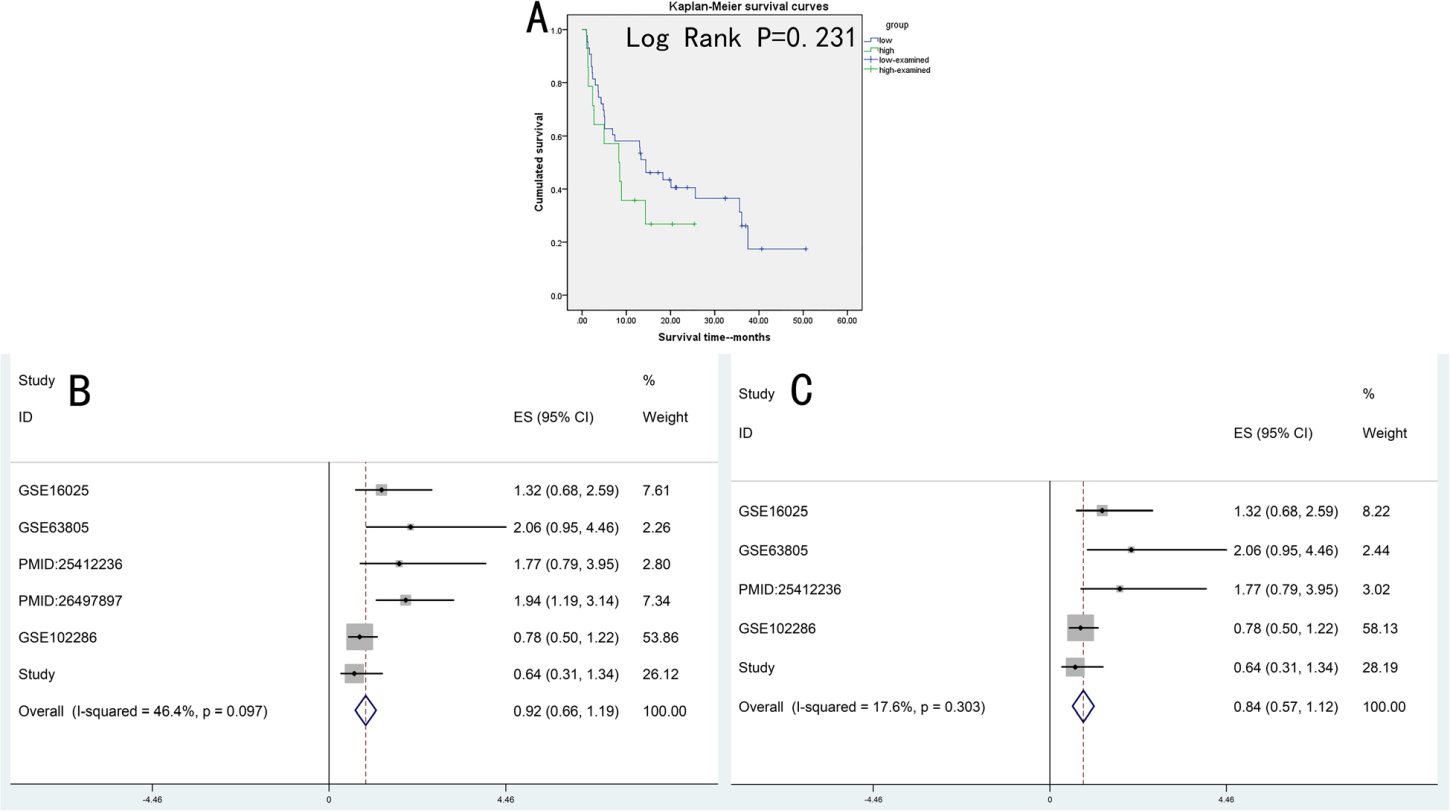
Kaplan-Meier curve of our study data and the prognostic analysis of miR-204-5p. **a**: High level of miR-204-5p seemed correlated with longer survival time, but the result was not statistically significant (P = 0.231). **b**. Forest plots of all research. **c**. Subgroup analysis of samples from tissues

The K-M plots of our RT-qPCR data indicated a correlation between the NSCLC survival rate and miR-204-5p expression, as patients with higher levels of miR-204-5p survived longer than those with lower expression, although the difference did not meet statistical significance (Log Rank *P* = 0.231) (Fig. 12a).

Only two publications were deemed eligible for prognostic assessment. The general information of 2 included references, 3 GEO datasets, and our study matched the required assessment conditions for a sum of 415 participants, as shown in Table 7. The HR and 95% CI were not included in the paper by Shi L [59], so they were calculated from the K-M survival curves, and the results with high statistical significance was considered in the selection for next step.

**Table 7:** Detailed information for miR-204-5p survival analysis. Annotation: HR, hazard ratio; LL, lower limit of the 95% confidence interval; UL, upper limit of the 95% confidence interval; OS, overall survival

ID	Author	Year	Country	Sample type	Citation	Cutoff	Method	Survival type	Sample size	HR	LL	UL
GSE16025	Raponi M	2009	USA	tissue	[44]	median	Univariate analysis	OS	61	1.322	0.675	2.590
GSE63805	Robles AI	2014	USA	tissue	[55]	median	Univariate analysis	OS	32	2.060	0.951	4.463
PMID:25412236	Shi L	2014	China	tissue	[59]	median	Kaplan–Meier analysis	OS	48	1.770	0.790	3.950
PMID:26497897	Guo W	2015	China	plasma	[15]	median	Univariate analysis	OS	126	1.936	1.193	3.143
GSE102286	Mitchell KA	2017	USA	tissue	[58]	median	Univariate analysis	OS	91	0.776	0.495	1.215
Current study	NR	NR	China	tissue	NR	median	Univariate analysis	OS	57	0.640	0.306	1.340

The results shown in Fig. 12b and c indicate that the use of miR-204-5p as an auxiliary prognostic risk factor for NSCLC patients is not possible at present, due to the lack of statistical significance in the prognostic meta-analysis (95% CI: 0.660 to 1.188), and a lack of large-scale investigations in plasma.

### Screening and validation of miR-204-5p target genes

In total, 4399 target genes were identified in at least six online predicted applications from miRwalk and 4371 up-regulated genes with FDR<0.05 were screened from TCGA. Subsequent analysis therefore focused on 541 over-active candidate genes from the intersection of miRwalk and TCGA.

The DAVID online tool identified 106 terms from the GO analysis and the top 3 most significantly enriched items associated with biological process (BP), cellular component (CC), and molecular function (MF) are listed in Table 8 (*P* < 0.05). The relevant target genes were chiefly involved in neuron projection, transcription factor activity, RNA polymerase II transcription regulation, extracellular matrix metabolism, and ion channel activity. In addition, 7 enriched pathways of KEGG analysis were collected from the same platform. As shown in Table 8, the top 3 signal pathways (P < 0.05) were connected with microRNAs in cancer, cell adhesion molecules (CAMs), and signaling pathways regulating pluripotency of stem cells.

**Table 8:** Top three items of GO and KEGG analysis. Annotation: GO, gene ontology; BP, biological process; CC, cellular component; MF, molecular function; KEGG, Kyoto Encyclopedia of Genes and Genomes

Category	ID	Term	Count	%	P value	Genes
BP	GO:0001764	neuron migration	13	0.015581	3.06E-06	PHOX2B, NDE1, SATB2, CDK5R1, CDK5R2, NAV1, SOX1, NTRK2, CELSR3, NEUROD4, DCX, FBXO45, PITX2
BP	GO:0051965	positive regulation of synapse assembly	8	0.009588	4.35E-04	SLITRK1, SRPX2, NTRK2, IL1RAP, EFNA5, TPBG, EPHB1, EPHB2
BP	GO:0008284	positive regulation of cell proliferation	17	0.020375	6.96E-04	CDC7, FGF5, HMX2, E2F3, RARG, PKHD1, SOX4, GREM1, EPHA1, GDNF, IL11, HDAC1, TFAP2B, POU3F2, EIF5A2, DPP4, DLG1
CC	GO:0043005	neuron projection	12	0.014382	2.91E-04	TENM4, TENM1, KIF5A, STMN2, SLC6A2, OPRK1, BCL11B, KIF5C, STMN4, GABBR2, DCX, CALB1
CC	GO:0005887	integral component of plasma membrane	38	0.045544	7.23E-04	GPR83, SLC5A3, SLC13A5, SLC20A2, SLC6A2, OPRK1, LRRC8D, GNRHR, CNGB3, SLC52A3, LGR4, EPHB1, EPHB2, EPCAM, ADRA2A, HCN3, HCN1, SLC12A7, GABRG2, CLCA2, RET, SLC6A17, MMP15, EPHA1, GRM1, SLC7A11, TIGIT, TENM4, EPHA7, SLC16A7, TMPRSS11D, TENM1, SLC6A8, SLC17A4, NTRK2, CLDN1, KCNH8, HAS3
CC	GO:0005667	transcription factor complex	12	0.014382	0.005459	E2F3, SATB2, BARX2, RARG, HNF1A, TRPS1, SIX1, TP63, POU3F2, TBL1X, TP73, PITX2
MF	GO:0005248	voltage-gated sodium channel activity	5	0.005993	5.84E-04	HCN1, SCN8A, SCN5A, HCN3, SCN4A
MF	GO:0005249	voltage-gated potassium channel activity	7	0.00839	8.94E-04	HCN1, KCNQ5, KCNH8, KCNA7, HCN3, CNGB3, KCNE4
MF	GO:0001077	transcriptional activator activity, RNA polymerase II core promoter proximal region sequence-specific binding	15	0.017978	0.001203	PHOX2B, FOXL2, SOX1, ONECUT2, SOX4, TP63, SIX2, HLTF, TP73, HOXC11, BCL11B, SIX1, TFAP2B, TFAP2A, POU3F2
KEGG	cfa05206	MicroRNAs in cancer	10	0.011985	0.005364	E2F1, DNMT3A, E2F3, WNT3, MMP9, IGF2BP1, TP63, CDK6, MMP16, HMGA2
KEGG	cfa04514	Cell adhesion molecules (CAMs)	8	0.009588	0.039758	TIGIT, SDC1, CLDN19, CLDN1, CNTNAP2, VCAN, NRXN1, CDH2
KEGG	cfa04550	Signaling pathways regulating pluripotency of stem cells	8	0.009588	0.04251	DVL3, FZD10, WNT3, HNF1A, INHBE, JARID2, NEUROG1, JAK3

**Fig. 13:**
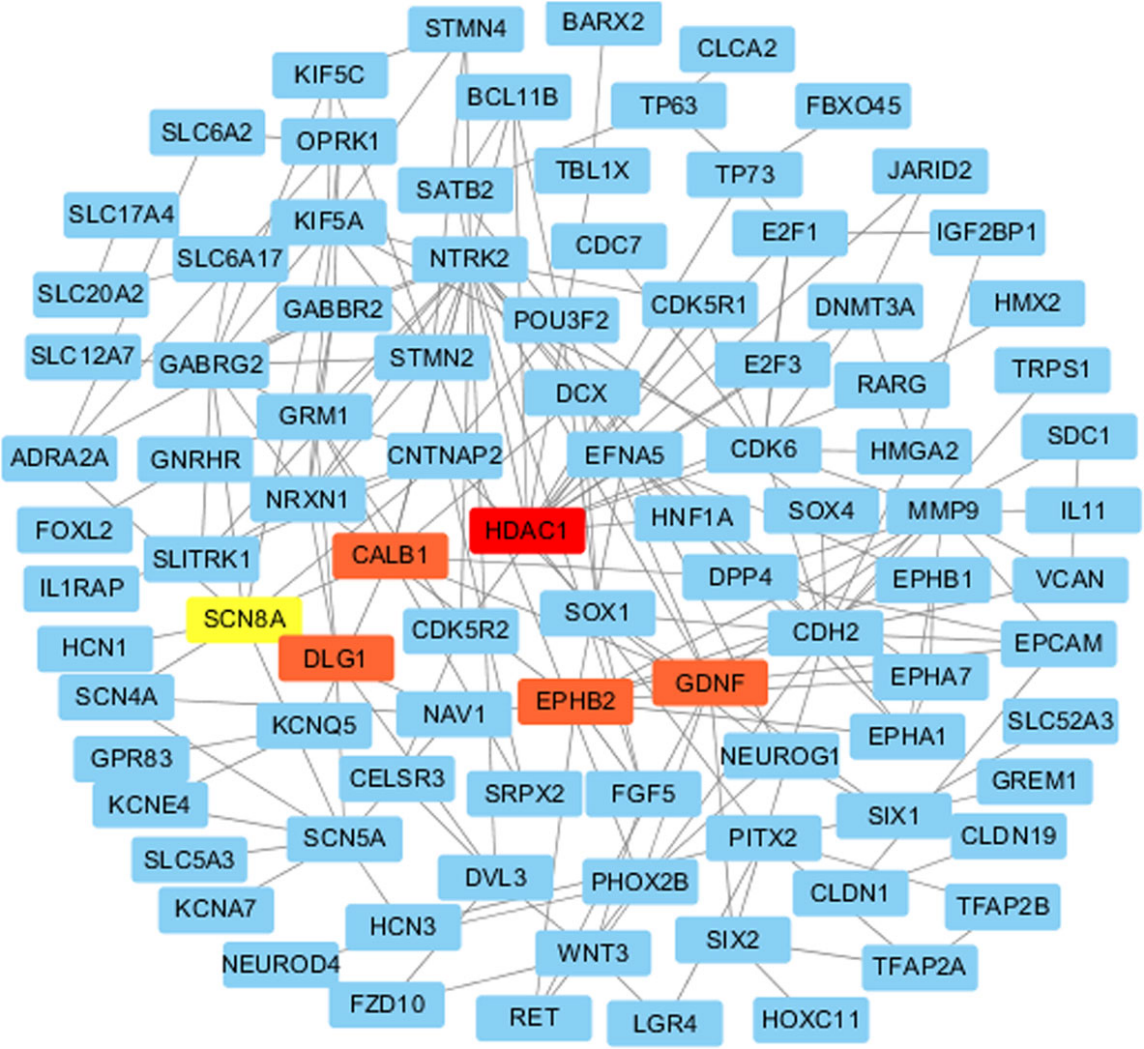
Protein -protein network of 117 hub genes for miR-204-5p

Taking the differences in genetic expression and function into account, the PPI network of 117 DEGs from the top three GO items and KEGG pathways was explored by STRING and visualized by Cytoscape to determine the interaction between the proteins encoded by candidate target genes. As Fig. 13 shows, the network consisted of 117 nodes and 130 edges. The top 6 proteins with the highest degrees of connectivity were HDAC1 (degree = 10), SCN8A (degree = 9), DLG1 (degree = 8), EPHB2 (degree = 8), GDNF (degree = 8) and CALB1 (degree = 8).

**Fig. 14:**
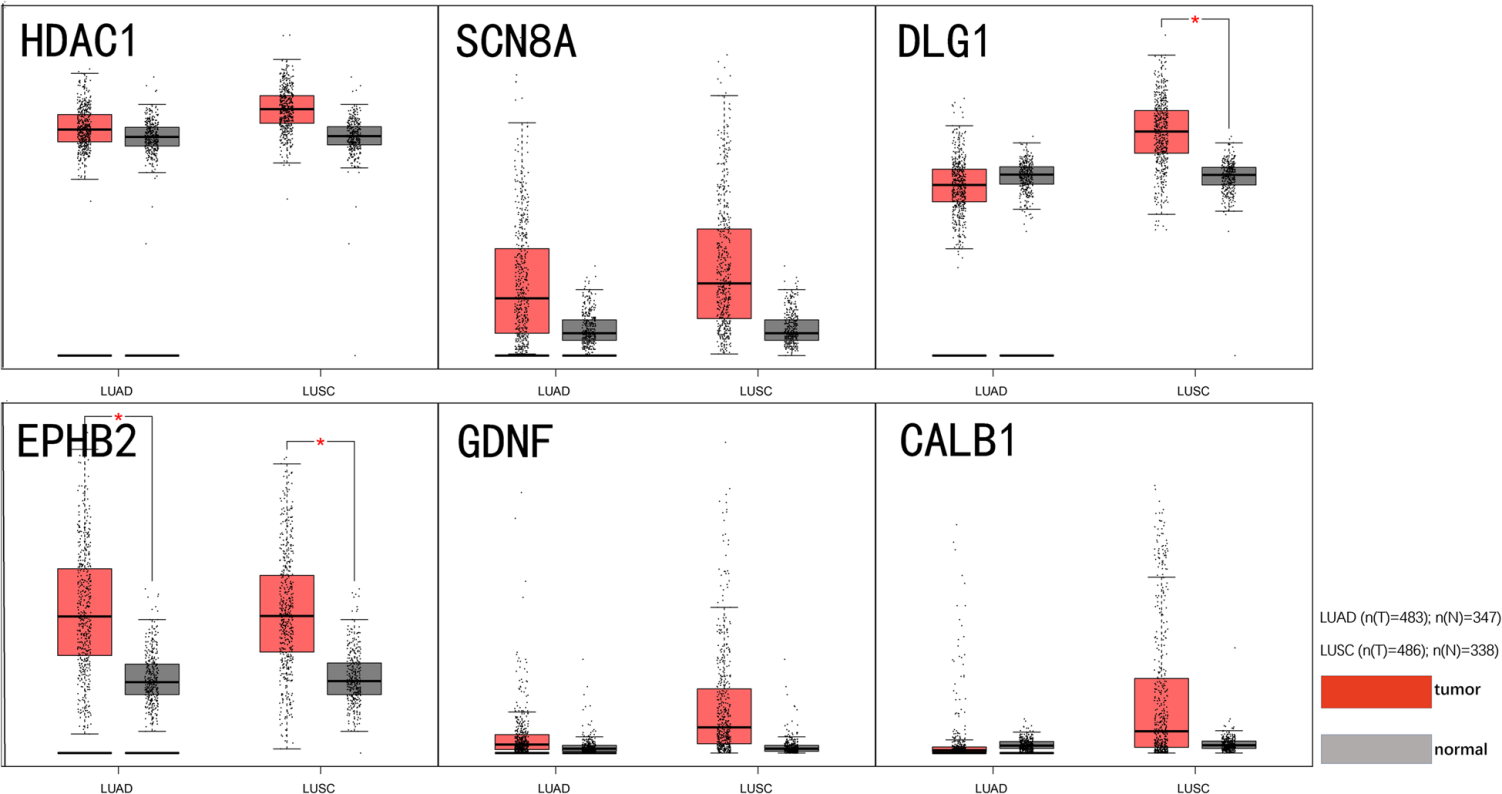
Scatter point plots of mRNA level for the six hub genes from GEPIA

**Fig. 15:**
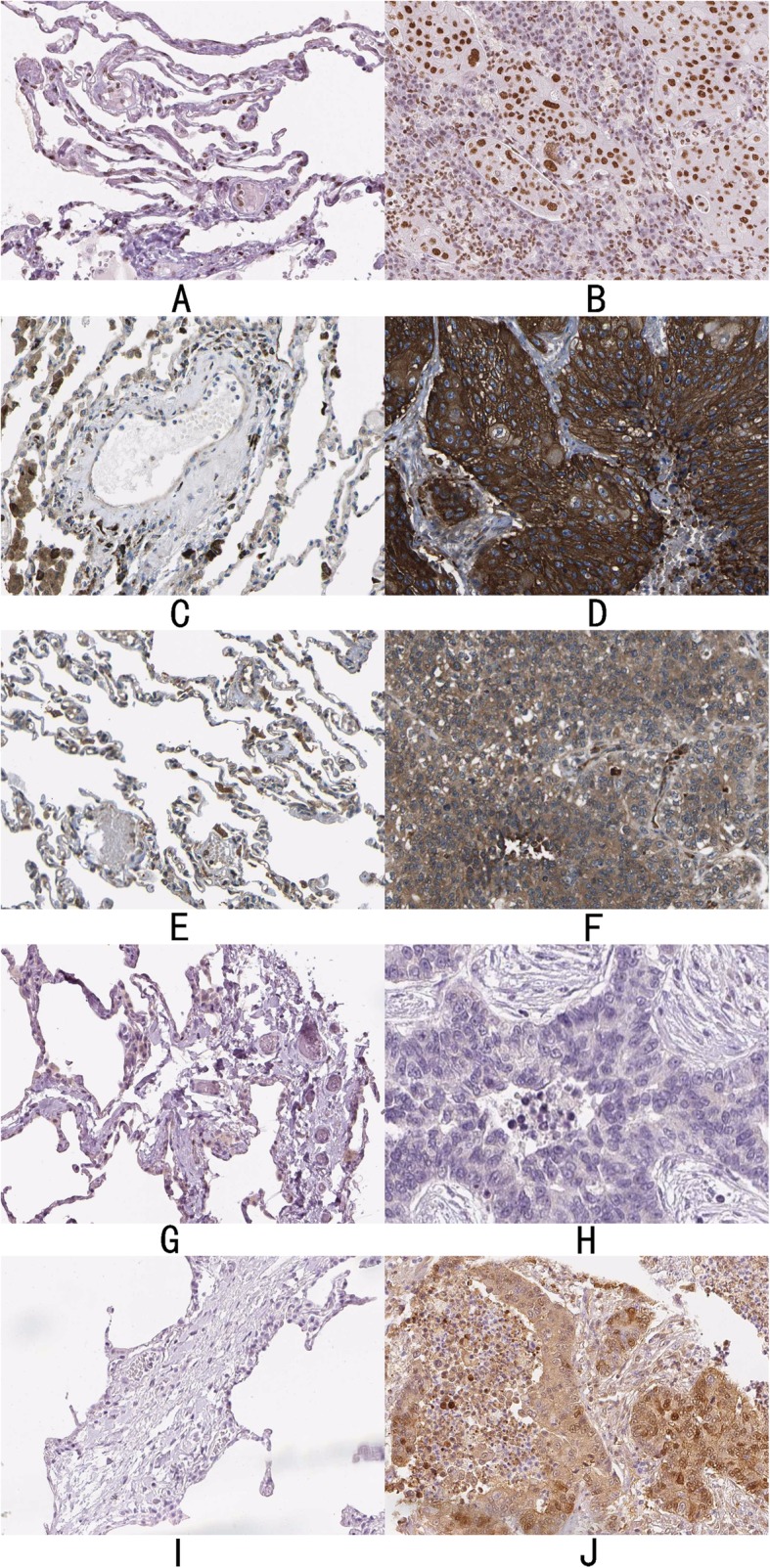
Protein expression variation of hub genes in non-small cell lung cancer (NSCLC) tissues and normal pneumocytes from THPA. **a** HDAC1 in normal pneumocytes (antibody CAB068191). **b** HDAC1 in NSCLC tissues (antibody CAB068191). **c** DLG1 in normal pneumocytes (antibody CAB016307). **d** DLG1 in NSCLC tissues (antibody CAB016307). **e** EPHB2 in normal pneumocytes (antibody CAB013647). **f** EPHB2 in NSCLC tissues (antibody CAB013647). **g** GDNF in normal pneumocytes (antibody CAB005210). **h** GDNF in NSCLC tissues (antibody CAB005210). **i**: CALB1 in normal pneumocytes (antibody HPA023099). **j** CALB1 in NSCLC tissues (antibody HPA023099). From the immunohistochemistry results of THPA, HDAC1, DLG1, EPHB2 and CALB1 trended to raise in NSCLC tissues, but GDNF was not apparently changed. Beyond that, no data was available for SCN8A

Scatter point plots from GEPIA indicated that expression of the six hub genes was elevated in NSCLC, and that EPHB2 had the most apparent variation. Of particular interest, DLG1 and GDNF showed a pronounced trend of over-expression in LUSC (Fig. 14). Besides no record about SCN8A, THPA confirmed a similar tendency for an increased expression of HDAC1, DLG1, EPHB2 and CALB1(Fig. 15), while GDNF was not apparently changed in either normal or lung cancer tissues; no data were available for SCN8A. In addition to GDNF (aliases ATF or ATF2) identified from the available literature [60], EPHB2 and DLG1 have been proposed as suitable targets of miR-204-5p. Further comprehensive investigations and systematic evaluations are needed to confirm this hypothesis because of small sample size of THPA and a lack of statistical analysis.

### TFs and the miR-204-5p regulatory network

**Fig. 16:**
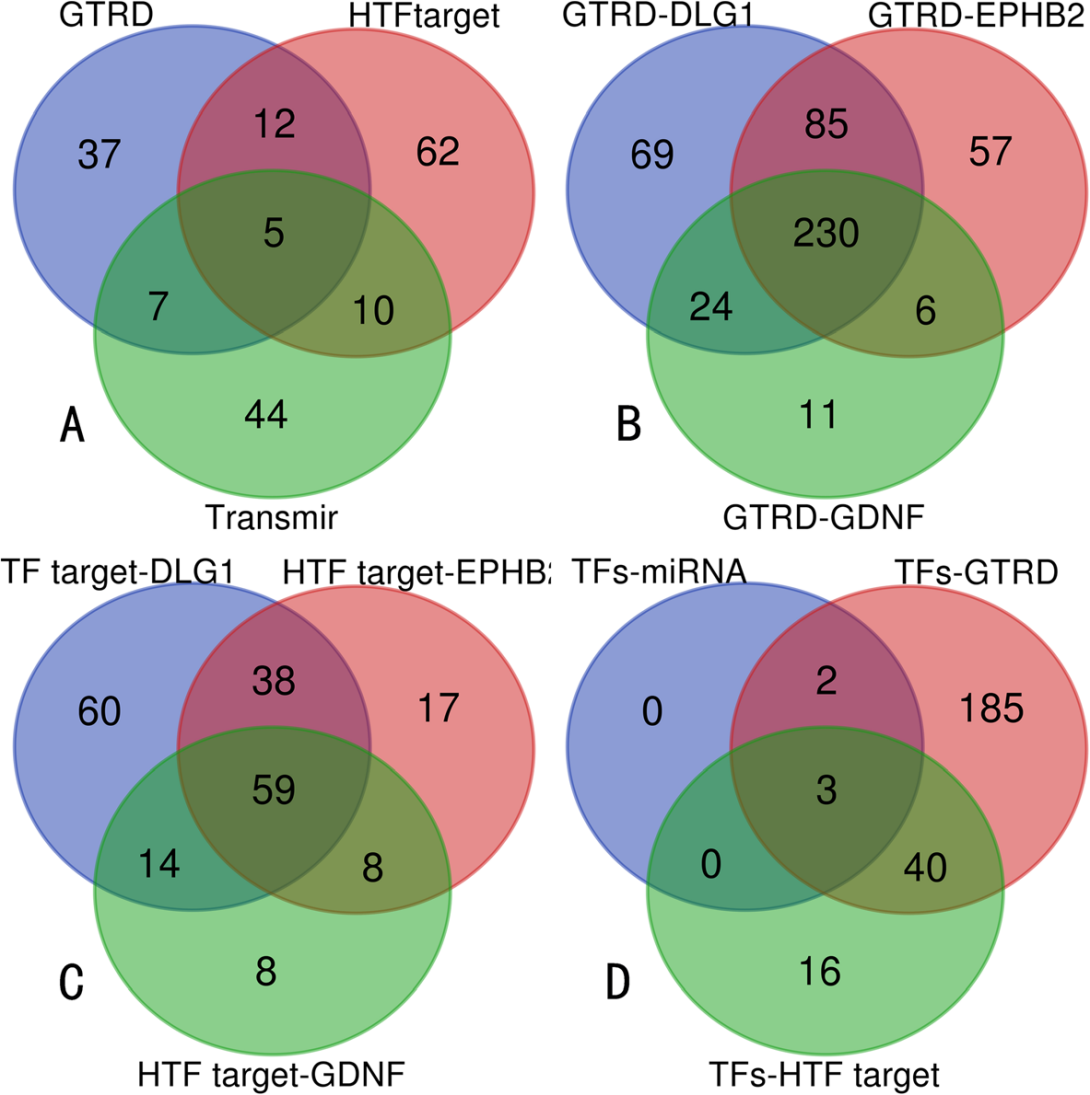
Venn charts for transcription factor screening. **a** Predicted transcription factors (TFs) of miR-204-5p from GTRD, HTF target and Transmir. **b** Predicted TFs of the three crucial hub genes from GTRD. **c** Predicted TFs of the three crucial hub genes from HTF target. **d** Predicted candidate TFs from coherent intersection of the foregoing three

**Fig. 17:**
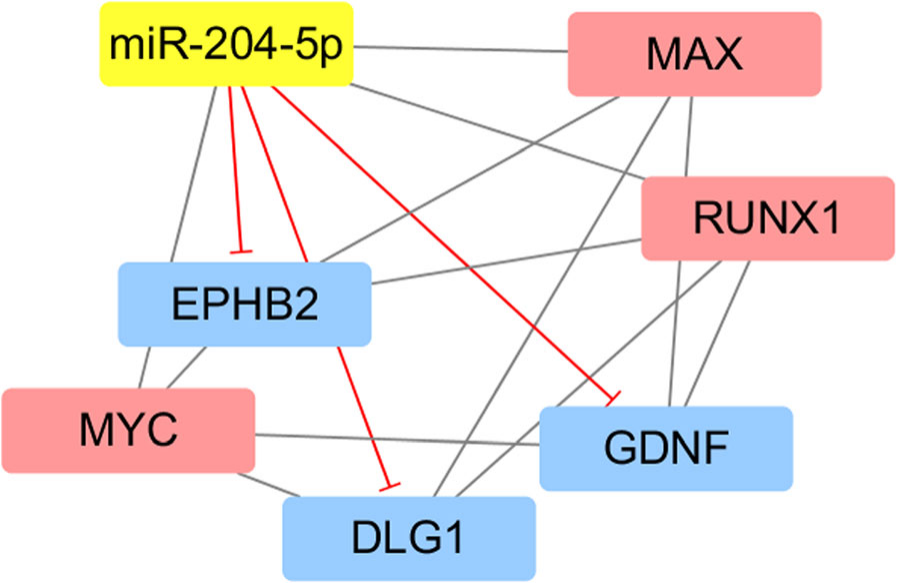
Relationships of miR-204-5p, genes and the predicted transcription factors (TFs). Apart from binding competition of miRNA towards hub genes, the combined ways of TFs with miR-204-5p and genes were still in doubt due to lack of database prediction, experimental proof and literature confirmation

In the present work, 61, 89, and 66 TFs related to miR-204-5p were obtained from GTRD, HTFtarget and Transmir, respectively. TF prediction was mainly matched examined for GDNF, DLG1, and EPHB2 since these genes were implicated as likely target genes. In total, GTRD and HTFtarget revealed 378 and 122 TFs of EPHB2, 408 and 171 TFs of DLG1, 271and 89 TFs with GDNF, respectively. The intersection outcome revealed MAX, MYC, and RUNX1 as the main TFs associated with miR-204-5p, GDNF, EPHB2, and DLG1(Fig. 16), while MAX was associated with miR-204-5p by TFBS prediction in JASPAR, which included some similar sequences in miR-204-5p, EPHB2, and GDNF. In addition, the putative TFBSs of MYC approached a certain degree of coincidence with MAX (Table 9). Binding competition of miRNA towards hub genes was confirmed by the miRwalk database and publication, but pairwise interactions of miR-204-5p with TFs and TFs interactions with hub genes could not be definitively constructed due to lack of database prediction, experimental proof and literature confirmation, so structural motifs of miR-204-5p networks could not be established (Fig. 17).

**Table 9:** The predicted transcription factors and the predicted sequences for miR-204-5p and the main hub genes

Gene	TF name	Score	Relative score	Start	End	Strand	Predicted sequence
miR-204-5p	MAX	6.92367	0.811791	21	30	+	TGACTCGTGG
DLG1	MAX	8.54191	0.861233	2277	2286	+	AAACAAGTGA
	RUNX1	7.92698	0.834755	2446	2456	+	TTATGAGGTAG
EPHB2	MAX	10.4915	0.928629	402	411	+	TCCACGTGGA
	MYC	11.9965	0.918300	401	412	+	ATCCACGTGGAG
GDNF	MAX	6.56373	0.800793	116	125	+	AGTCTCGTGC
	MYC	6.37509	0.800941	116	127	+	AGTCTCGTGCTC
	RUNX1	10.8526	0.910532	1943	1953	+	AGTTGTGGTTT

## Discussion

The data presented here verified the decrease in miR-204-5p expression in NSCLC by comprehensive analysis of RT-qPCR, microarrays, sequencing data, and publications and revealed an obvious decrease in cancerous tissues and the LUAD subtype. An auxiliary role for miR-204-5p was also identified by in NSCLC, particularly in tissues and LUAD, which was verified by the meta-analysis. Unfortunately, the prognostic implications for miR-204-5p were weak and showed no statistical significance in the meta-analysis, due to a shortage of large-scale investigations. Nevertheless, the information provided by GO annotation and KEGG analysis indicated that the target genes of miR-204-5p were associated with neuron projection, transcription factor activity, RNA polymerase II transcription regulation, extracellular matrix (ECM) metabolism, and ion channel activity, as well as were connected with microRNAs in cancer, cell adhesion molecules (CAMs) and signaling pathways regulating pluripotency of stem cells. Three of the six hub genes, GDNF, EPHB2 and DLG1, were selected for continued research due to their distinct characteristics in NSCLC. TF prediction revealed speculatively functional relations of MAX, MYC, and RUNX1 between miR-204-5p and these three genes, although only MAX demonstrated the TFBS sequences connected with miR-204-5p upon further query.

At present, miR-204-5p has aroused considerable interest in cancer research for its dual function as an oncogene and tumor suppressor [14]. MiR-204-5p is clearly attenuated in NSCLC, and its expression is negatively linked with tumor size, clinical stage, and metastasis [28–30, 59, 61]. Downregulation of miR-204 occurs in part due to its hypermethylation in the promoter region [59]. Elevated expression of miR-204-5p depresses NSCLC migration and invasion by targeting Janus kinase 2 (JAK2) [17], restrains proliferation of NSCLC cells by regulating SIX homeobox 1 (SIX1) and attenuates LUAD angiogenesis potentially by JAK2-signal transducer and activator of transcription 3 (JAK2-STAT3) pathway [16]. In addition, miR-204-5p serves as a cancer suppressor gene by modulating oncogenic Wnt/FZD signaling pathways [62], inhibiting NUAK family kinase 1 (NUAK1) in NSCLC [59], and mediating a long-noncoding RNA (lncRNA) MALAT1 effect on the epithelial-to-mesenchymal transition (EMT) and cells invasion [63]. Our results confirmed the downregulation of miR-204-5p expression in NSCLC and revealed a constant level of decline in LUAD.

The integrative meta-analysis also indicated a promising role for miR-204-5p for NSCLC screening, as did subgroup SROC curves, even though the sample origins were different. The variation in miR-204-5p expression in tissues was helpful in diagnosis of LUAD than of LUSC. Considering the invasive work, high cost, and cumbersome procedure of tissue biopsy, analysis of blood circulating miR-204-5p was considered an attractive screening indicator. However, low sensitivity, high specificity, and small sample sizes of the currently available data mean that more research and detailed profiling at all levels are needed to provide information to confirm the effectiveness of blood screening.

The correlation between the low miR-204-5p and high risk of death in patients with NSCLC [15, 59] was evident in our study but failed to reach statistical significance. The subsequent integrative meta-analysis was conducted to gain insights into the potential usefulness of miR-204-5p in NSCLC prognosis, but the data from the literature and from the present study regarding the ability of miR-204-5p to predict survival times of patients with NSCLC are conflicting. Consequently, no conclusion can be made in terms of miR-204-5p for NSCLC, at least for now.

The GO analysis indicated that the estimated target genes were mainly enriched in neuron projection, transcription factor activity, RNA polymerase II transcription regulation, ECM metabolism and ion channel activity, suggesting a potential involvement of miR-204-5p in the molecular function and signal modulation associated with NSCLC biological processes. The KEGG pathway analysis indicated that some of candidate genes were participated in microRNAs in cancer, CAMs, and signaling pathways regulating pluripotency of stem cells. Like other miRNAs, miR-204-5p plays an indispensable role in cancer proliferation, migration, and metastasis by regulating the tumor microenvironment, such as ECM structure and CAM metabolism [64]. Cancer stem cells (CSCs) attain stemness by complicated processes and signaling pathways, such as JAK-STAT, nuclear factor kappa B, Sonic hedgehog, transforming growth factor beta, Wnt/β-catenin, and PI3K/AKT [65, 66]. Many miRNAs take part in processes that maintain a balance between differentiation and quiescence of pulmonary CSCs, adjust the tumor microenvironment and affect cell cycle progression via regulation of these signaling pathways [67]. Consequently, miR-204-5p and its target genes could serve as important determinants of NSCLC pathogenesis and development.

Continued investigation of the hub genes involved in GO enrichment and KEGG Pathway analysis identified six leading relevant genes that were screened out due to binding to 3’UTR of miRNA. However, only GDNF has been investigated for a direct relationship with miR-204-5p in NSCLC [60].

GDNF, also called ATF or ATF2, is a well-characterized oncogene that promotes tumor growth, invasion, and metastasis, in addition to tumor microenvironment alterations [68]. GDNF expression occurs high level in NSCLC, though a significant difference exists with regard to factors such as race, gender, age, smoking status, and histologic subtype [69]. GDNF is upregulated at the transcript level in LUSC [70], and is hypermethylated in tumor tissues [71]. It also facilitates demethylation of the fibromodulin promoter and promotes subsequent angiogenesis in human glioblastomas [72]. Nerve-derived GDNF increases programmed death ligand 1 (PD-L1) levels in head and neck squamous cell carcinoma cells by activating the JAK2-STAT1 signaling pathway, which in turn promotes the evasion of cancer cells from immune system surveillance in the nerve-cancer microenvironment [73]. Recent research in colorectal cancer (CRC) has indicated that miR-196a-5p exerts its function in cell proliferation and migration by regulating GDNF expression [74], while miR-451 influences drug resistance in renal cell carcinoma by targeting GDNF [75]. GDNF is also targeted and regulated by miR-204-5p which inversely affects GDNF mRNA and protein levels, to inhibit NSCLC growth, migration, and cell cycle alteration and promote apoptosis [60]. Therefore, the interaction between miR-204-5p and GDNF appears to be critical in the development and progression of NSCLC and requires thorough research.

DLG1 is a vital participant in the control of cellular processes like polarity, proliferation and migration, so its dysregulation and mutation give rise to pathologies that include oncogenic processes [76]. DLG1 is mainly identified as a tumor suppressor, since overexpression is observed early in the onset of cervical cancer (CeCa) [77] and elevated DLG1 promotes intestinal tumorigenesis [78], predicts poor prognosis in people with CRC [79] and increases the invasiveness of NSCLC cell lines [80]. Increased phosphorylation of the DLG1 SH3-Hook region promotes interaction with the PDZ ligand of PKCα and accelerates cell migration [80]. The lncRNA DLG1-AS1 acts as a competitive inhibitor that influences the activity of miR-107 on its target gene ZHX1, thereby inducing cancer cell proliferation [81]. Moreover, DLG1 deficiency results in incorrect spindle polarity and a delay in cells transiting orientation [78], which disrupts cellular structure and distribution [82]. Interestingly, DLG1 protein levels are significantly lower in NSCLC and hepatocellular carcinoma (HCC) than in the corresponding normal tissues [83, 84], but are nearly undetectable in poorly differentiated stages of colon adenocarcinoma [85], in contrast to our findings and the existing literature. One possible reason is that DLG1 dysregulation in advanced tumor progression or in more malignant forms depends on its spatial/temporal distribution. Future research should focus on this possibility.

A series of studies have reported a direct correlation between EPHB2 expression and numerous human malignancies, including NSCLC. EPHB2 activates bidirectional signaling cascades and its upregulation predicts poor survival in LUAD [86], CRC [87], breast cancer [88] and malignant mesothelioma [89]. One study indicated that EPHB2 enhances cellular growth, migration and invasion in CeCa by a competitive inhibition that counteracts the miR-204 effect on cell cycle arrest, Bax overexpression and PI3K/AKT signaling pathway deactivation via competitive inhibition [90]. Expression of miRNAs also significantly suppresses EPHB2 expression, resulting in a decrease of tubulogenesis and angiogenesis [91, 92]. EPHB2 affects cell viability in medulloblastoma in part by promotion of the G2/M phase of the cell cycle [93]. Activation of EPHB2 promotes the progression of cutaneous squamous cell carcinoma cells by accelerating the production of invasive proteinases like MMP13 and MMP1 [94]. Nevertheless, some publications highlight EPHB2 declines in CRC, which was supposedly attributable to EMT modulation [95] and epigenetic modification of promoter [96, 97]. Future research could identify where whether specific differences in the interaction of EPHB2 and miR-204-5p are associated with NSCLC.

As discussed above, the function of miR-204-5p in NSCLC is also influenced by TFs as well, but an unanswered question is whether TFs regulate miR-204-5p or whether TFs could be adjusted or controlled by this miRNA in some way. Although great achievements have been made in understanding the biological behavior of miR-204-5p and its mRNA targets, integrative analysis of miRNA-TF-gene regulatory networks is still needed, as TFs are undoubtedly involved in pulmonary cancer initiation, progression, dissemination, recurrence, and even drug resistance [98]. At least ten kinds of TF-miRNA synergistic regulatory networks apparently function in NSCLC [99]. In addition to combining with and regulating its target genes, miR-204-5p also attenuates some angiogenic inducers like hypoxia inducible factor-1α (HIF-1α) to impair angiogenesis in LUAD [16]. Another study has demonstrated a dependence of miR-204-5p level on promoter hypermethylation and support by positive feedback of three TFs, c-MYB, ETS1 and RUNX2 [100]. Osterix, a transcription factor that is essential and specific for osteogenesis, coordinately modulates miR-204-5p and its endogenous competitors, as well as ultimately establishing a feed-forward loop (FFL) ultimately [101]. Activation of STAT3 suppresses miR-204-5p activities, in turn affecting proliferation and apoptotic resistance in human pulmonary arterial hypertension and nasopharyngeal carcinoma [102, 103]. A positive FFL between Hepatitis B virus, miR-204-5p, and STAT3 appears to contribute to HCC incidence [104].

Other work has suggested that miR-204-5p reciprocally represses TrkB expression; however, TrkB expression noticeably increases JAK2 and STAT3 phosphorylation. The phospho-STAT3 then directly binds to promoter sequence of miR-204-5p, resulting in increased clonogenic proliferation, migration and invasion in endometrial carcinoma, via a feed-backward-loop (FBL) motif of TF-miRNA-target gene [105]. The activity of the IL-6R/STAT3/miR-204-5p FBL also leads to chemosensitivity [106]. The possibility exists that, at molecular level, the cellular activities involved in NSCLC progression, including tumor cell proliferation, differentiation, invasion, apoptosis, recurrence, and even drug resistance, are associated with downregulation of miR-204-5p and account for the direct upregulation of its target genes as well as unidirectional or bidirectional activation of TFs.

Among the three TFs expected to take part in miR-204-5p networks, MAX forms a dimer-complex system of transcriptional regulation with other family members, which include MYC, Mad and Mxi1, and is implicated in cell proliferation, differentiation and apoptosis [107]. The sequence similarity in MAX and MYC TFBS in the current work was predicted based on the existence of the multiprotein complex. An increased expression of miR-22 in leukemia cells reduces the MAX expression level, blocking cell cycle progression at the G1 phase [108]. MAX expression in HCC activates Linc00176, which is a competing endogenous lncRNA (ceRNA) of tumor-suppressive miRNA, resulting in cell cycle acceleration and reduction of apoptosis by reducing the levels of miR-9 and miR-185 [109]. In CRC, a MAX/MYC heterodimer induced by elevated HIF-2α mediates transcriptional repression of hypoxia-related miR-15-16, leading to tumor angiogenesis and hematogenous metastasis by further loss of post-transcriptional restriction towards fibroblast growth factor-2 [110]. MYC, also known as MYCC and c-Myc, is frequently amplified in numerous human cancers via transcriptional regulation of specific target genes, including miRNA and lncRNA [111]. MiR-296 − 3p directly targets PRKCA to impair FAK-Ras-MYC signaling, thereby accelerating its own transcription in a FBL that obstructs the EMT signal and progression through the cell cycle, following suppression of cell proliferation, metastasis and chemosensitivity in LUAD [112]. Another study has suggested that miR-342-3p is capable of indirectly adjusting MYC by directly repressing E2F1, a MYC-collaborating molecule [52]. In breast tumor, MYC expression correlates positively with miR-203b-3p and miR-203a-3p but negatively with BCL2L1 expression, resulting in formation of a TF-FFL [113]. MIR7-3HG restrains MYC dephosphorylation by downregulation of AMBRA1 to form a positive feedback loop for its own expression and further contributing significantly to autophagic control [114].

As a crucial hematopoietic transcription factor, RUNX1 is well-documented in chromosomal translocations and in several types of carcinogenesis processes [115]. RUNX1 is positioned in the center of miRNA circuits relevant for malignant hematopoiesis in transcriptional programs [116, 117]. A RUNX1-microRNA-139-HCP5 axis shows a positive FBL for mediating the tumor-suppressive effects of glioma cells [118]. A miR-18a-RUNX1-ZO-1 regulatory network also increases the permeability of the blood-tumor barrier (BTB), thereby providing novel potential targets for drug transportation across the BTB as an attractive strategy for glioma treatment [119]. By binding to the miRNA promoter, RUNX1 increases the transcriptional level of miR-27a in breast cancer and concomitantly the decreases expression of ZBTB10, a direct target gene of miR-27a, to promote endothelial differentiation and subsequent angiogenesis and tumor metastasis [120]. Conversely, reduced expression of Runx1 in breast cancer cells leads to elevated expression of both pre-miR-378 and PPARGC1B, which is a host gene of miR-378, to create a FBL on that reduces cell migration and invasion [121]. The miR-204-5p circuits and its hub genes and TFs still await identification, but TFBS prediction was capable of offering fresh perspectives, and likewise, assisting in new theoretical insights into potential regulatory mechanisms. Based on the available data, MAX would appear likely to be a vital TF involved in miR-204-5p-mRNA interactions, since it was the only assumed attachment that focused on the upstream region of genetic sequences in the current work.

This study had drawbacks and limitations. One limitation is that the findings indicate a great heterogeneity between the data sources, which were then explored via a random effects model and subgroup meta-analyses. The trial quality was also generally poor due to heterogeneity that remained above 50%. One possible cause of the statistical heterogeneity is that the data were generated using different sources, operating protocols, and detection metrics. Univariate survival analysis is also the only appraisal method for determining the prognostic significance of miR-204-5p. A carefully designed evaluation system should be developed to provide a more in-depth assessment of this issue. In particular, time limits and tight budgets have prevented a satisfactory generation of experimental proof to validate the function of miR-204-5p regulatory networks with the target genes and TFs. In addition, the biological progression and molecular regulation of NSCLC is complicated, so other mechanisms mediated by miRNA circuits should also be addressed with in-depth research.

## Conclusion

As the most frequent type of pulmonary cancer, NSCLC deserves more effort in achieving the goal of early detection and timely treatment. The findings presented in the current research demonstrated an attenuation of miR-204-5p expression in NSCLC, this decrease was more frequently observed in cancerous tissues and in the LUAD subtype and was, in part, helpful for diagnosis. The activities of miR-204-5p as an anti-oncogene were induced by its regulatory axes or circuits with target genes and TFs that participated in specific genetic pathways and biological processes.

## Data Availability

Data and material will be available on reasonable request.

## Abbreviations

95% CI: 95% confidence interval
DEGs: Differentially expressed genes
GEO: Gene expression omnibus
GO: Gene ontology
HR: Hazard ratio
KEGG: Kyoto encyclopedia of genes and genomes
K-M curve: Kaplan-Meier curve
LUAD: Lung adenocarcinoma
LUSC: Lung squamous cell carcinoma
MiRNA: microRNA
NSCLC: Non-small cell lung cancer
ROC: Receiver operating characteristic
RT-qPCR: Real-time quantitative PCR
SMD: Standard mean deviation
TCGA: The cancer genome atlas
TF: Transcription factor
